# Glial reactivity and T cell infiltration in frontotemporal lobar degeneration with tau pathology

**DOI:** 10.1093/brain/awad309

**Published:** 2023-09-13

**Authors:** Iain J Hartnell, Declan Woodhouse, William Jasper, Luke Mason, Pavan Marwaha, Manon Graffeuil, Laurie C Lau, Jeanette L Norman, David S Chatelet, Luc Buee, James A R Nicoll, David Blum, Guillaume Dorothee, Delphine Boche

**Affiliations:** Clinical and Experimental Sciences, Faculty of Medicine, University of Southampton, Southampton SO16 6YD, UK; Clinical and Experimental Sciences, Faculty of Medicine, University of Southampton, Southampton SO16 6YD, UK; Clinical and Experimental Sciences, Faculty of Medicine, University of Southampton, Southampton SO16 6YD, UK; Clinical and Experimental Sciences, Faculty of Medicine, University of Southampton, Southampton SO16 6YD, UK; Clinical and Experimental Sciences, Faculty of Medicine, University of Southampton, Southampton SO16 6YD, UK; Clinical and Experimental Sciences, Faculty of Medicine, University of Southampton, Southampton SO16 6YD, UK; Clinical and Experimental Sciences, Faculty of Medicine, Sir Henry Wellcome Laboratories, University of Southampton, Southampton O16 6YD, UK; Histochemistry Research Unit, Clinical and Experimental Sciences, Faculty of Medicine University of Southampton, Southampton SO16 6YD, UK; Biomedical Imaging Unit, University Hospital Southampton NHS Trust, Southampton SO16 6YD, UK; University of Lille, Inserm, CHU Lille, UMR-S1172—Lille Neurosciences and Cognition, Lille 59045, France; Alzheimer and Tauopathies, LabEX DISTALZ, Lille 59000, France; Clinical and Experimental Sciences, Faculty of Medicine, University of Southampton, Southampton SO16 6YD, UK; Department of Cellular Pathology, University Hospital Southampton NHS Trust, Southampton SO16 6YD, UK; University of Lille, Inserm, CHU Lille, UMR-S1172—Lille Neurosciences and Cognition, Lille 59045, France; Alzheimer and Tauopathies, LabEX DISTALZ, Lille 59000, France; Clinical and Experimental Sciences, Faculty of Medicine, University of Southampton, Southampton SO16 6YD, UK; Sorbonne Université, Inserm, Centre de Recherche Saint-Antoine, CRSA, Immune System and Neuroinflammation Laboratory, Hôpital Saint-Antoine, Paris 75012, France; Clinical and Experimental Sciences, Faculty of Medicine, University of Southampton, Southampton SO16 6YD, UK

**Keywords:** frontotemporal dementia, tau, microglia, astrocytes, T lymphocytes

## Abstract

Frontotemporal lobar degeneration with tau (FTLD-tau) is a group of tauopathies that underlie ∼50% of FTLD cases. Identification of genetic risk variants related to innate/adaptive immunity have highlighted a role for neuroinflammation and neuroimmune interactions in FTLD. Studies have shown microglial and astrocyte activation together with T cell infiltration in the brain of THY-Tau22 tauopathy mice. However, this remains to be confirmed in FTLD-tau patients. We conducted a detailed post-mortem study of FTLD-tau cases including 45 progressive supranuclear palsy with clinical frontotemporal dementia, 33 Pick’s disease, 12 FTLD-MAPT and 52 control brains to characterize the link between phosphorylated tau (pTau) epitopes and the innate and adaptive immunity. Tau pathology was assessed in the cerebral cortex using antibodies directed against: Tau-2 (phosphorylated and unphosphorylated tau), AT8 (pSer202/pThr205), AT100 (pThr212/pSer214), CP13 (pSer202), PHF1 (pSer396/pSer404), pThr181 and pSer356. The immunophenotypes of microglia and astrocytes were assessed with phenotypic markers (Iba1, CD68, HLA-DR, CD64, CD32a, CD16 for microglia and GFAP, EAAT2, glutamine synthetase and ALDH1L1 for astrocytes). The adaptive immune response was explored via CD4^+^ and CD8^+^ T cell quantification and the neuroinflammatory environment was investigated via the expression of 30 inflammatory-related proteins using V-Plex Meso Scale Discovery.

As expected, all pTau markers were increased in FTLD-tau cases compared to controls. pSer356 expression was greatest in FTLD-MAPT cases versus controls (*P <* 0.0001), whereas the expression of other markers was highest in Pick’s disease. Progressive supranuclear palsy with frontotemporal dementia consistently had a lower pTau protein load compared to Pick’s disease across tau epitopes. The only microglial marker increased in FTLD-tau was CD16 (*P* = 0.0292) and specifically in FTLD-MAPT cases (*P* = 0.0150). However, several associations were detected between pTau epitopes and microglia, supporting an interplay between them. GFAP expression was increased in FTLD-tau (*P* = 0.0345) with the highest expression in Pick’s disease (*P* = 0.0019), while ALDH1L1 was unchanged. Markers of astrocyte glutamate cycling function were reduced in FTLD-tau (*P* = 0.0075; Pick’s disease: *P* < 0.0400) implying astrocyte reactivity associated with a decreased glutamate cycling activity, which was further associated with pTau expression. Of the inflammatory proteins assessed in the brain, five chemokines were upregulated in Pick’s disease cases (*P* < 0.0400), consistent with the recruitment of CD4^+^ (*P* = 0.0109) and CD8^+^ (*P* = 0.0014) T cells. Of note, the CD8^+^ T cell infiltration was associated with pTau epitopes and microglial and astrocytic markers. Our results highlight that FTLD-tau is associated with astrocyte reactivity, remarkably little activation of microglia, but involvement of adaptive immunity in the form of chemokine-driven recruitment of T lymphocytes.

## Teaser

Hartnell *et al.* report that the neuroinflammation and neuroimmune interactions that characterize frontotemporal lobar degeneration with tau involve both innate and adaptive immunity, with the presence of astrocyte reactivity, limited microglial activation, and chemokine-driven recruitment of T lymphocytes.

## Introduction

Frontotemporal dementia (FTD) encompasses neurodegenerative diseases typified by behavioural/personality changes and language impairments. These symptoms result from neurodegeneration in the frontal and temporal lobes, termed frontotemporal lobar degeneration (FTLD). FTLD is further subclassified according to the nature of the protein aggregates that accumulate in the disease, including tau (∼40–45% of cases), TAR DNA-binding protein-43 (TDP43) and FUS.^1-3^

FTLD-tau diseases are characterized by tau hyperphosphorylation (pTau) and aggregation within cells. This occurs sporadically in FTLD-tau diseases such as Pick’s disease (PiD) and progressive supranuclear palsy (PSP), or due to a genetic mutation in *MAPT*, the gene encoding for tau (FTLD-MAPT). There are various known differences in tau pathology between the diseases of the FTLD-tau spectrum, including the protein structure (the number of repeats of the microtubule-binding domain) and neuropathological features. PSP features include neurofibrillary tangles in neurons and tau-positive tufted astrocytes. In contrast, PiD is uniquely associated with spherical tau inclusions in neurons, called Pick bodies, with other features displayed being Pick cells/ballooned neurons and tau-positive astrocytes, which are noted as being ramified in structure.^4,5^ The pattern of tau phosphorylation in tauopathies has been reported previously in small cohorts of FTLD-tau (*n* = 3–8) using a semi-quantitative scale,^6^ but it remains to be confirmed whether there are any disease-specific differences in the amount of tau.

Genetic, biomarker and experimental studies suggest that neuroinflammatory responses, mediated by microglia and astrocytes, play a complex role in FTLD-tau pathophysiology.^7^ Microglia, the key mediators of innate immunity in the brain, display critical physiological functions in maintaining homeostasis by clearing extracellular debris, refining neuronal circuitry, contributing to plasticity and producing neurotrophic factors. They exhibit a ramified morphology with fine processes, which survey the microenvironment for damage- or pathogen-associated molecular patterns. Upon activation, they acquire an amoeboid shape and migrate towards sites of injury, where they clear damaged tissue via phagocytosis. Microglia propagate the neuroinflammatory response through the release of cytokines, such as IL1α, IL1β, TNFα, TGFβ and various chemokines.^8^ Astrocytes have critical roles in neurotransmitter cycling, metabolic and trophic support to neurons and are key components of the blood–brain barrier. In response to CNS injury or infection, astrocytes become reactive, undergoing ‘astrogliosis’^9^ defined by a spectrum of changes in morphology, function and number^10^ that occur in response to pro-inflammatory cytokine signals.

Pre-clinical models of tau pathology reported activation of microglia and astrocytes together with upregulation of pro-inflammatory cytokines^11-14^ and post-mortem studies identified microglial activation in human tauopathies.^15,16^ Evidence supports the concept that neuroinflammation might precede tau pathology, as observed in mice^17^ and in a human TSPO-PET study,^18^ consistent with the glial cell activation identified in several neurodegenerative conditions.^19^

In addition to innate immune responses in the brain, cellular adaptive immunity may play an instrumental role in neurodegenerative diseases, with T-cell infiltration observed in Alzheimer’s disease, Parkinson’s disease, dementia with Lewy Bodies, as well as other causes of dementia and aged controls.^20-22^ In THY-Tau22 transgenic mice, CD8^+^ T cells infiltrate the hippocampus, in association with upregulation of T-cell attracting chemokines (CCL3, CCL4 and CCL5),^14^ also confirmed in three FTLD-MAPT patients. However, whereas most studies addressing the link between tau pathology and immune responses in the brain comes from experimental models, this association in human FTLD-tau patients remains ill-defined.

Here we conducted a detailed post-mortem study in a large cohort of FTLD-tau cases and control brains to characterize the link between phosphorylated tau epitopes and innate and adaptive immunity.

## Materials and methods

### Case selection

Cases were sourced from five UK brain banks: the MRC London Neurodegenerative Diseases Brain Bank (*n =* 27), the Manchester Brain Bank (*n =* 56), the Newcastle Brain Tissue Resource (*n =* 18), the Oxford Brain Bank (*n =* 15) and the South West Dementia Brain Bank (*n =* 26). The cohort was established based on the primary neuropathological diagnoses of PiD (*n =* 33), PSP with clinical FTD (PSP, *n =* 45) or FTLD-MAPT (IVS10 + 16, *n =* 11, IVS10 + 12, *n =* 1). Controls (*n =* 52) were selected on the neuropathological diagnosis by the brain banks reported as control, normal ageing, no abnormality detected or age changes only and on a basis of a tau-based Braak Stage of <3. They were matched for post-mortem delay, age and gender where possible (Supplementary Table 1). Identification of the cases will be provided on request to the corresponding authors including full neuropathological assessment for each case available on the UK Brain Bank Network database. The middle frontal gyrus (Brodmann area 9) was investigated. Formalin-fixed paraffin-embedded tissue from the 142 cases was acquired for the immunohistochemistry. Frozen tissue from the same area, in the contralateral hemisphere, was available for 100 of these cases (Supplementary Table 1), excluding the FTLD-MAPT cases, and was utilized for the inflammatory protein multiplex assay.

#### Ethical approval

The study was covered by the following ethical approvals: (i) MRC London Neurodegenerative Diseases Brain Bank (Committee for Wales, REC reference: 18/WA/0206); (ii) Manchester Brain Bank (REC Reference 19/NE/0242); (iii) Newcastle Brain Tissue Resource (Newcastle and North Tyneside 1 Research Ethics Committee, REC reference: 09/H0906? 52 + 5); (iv) Oxford Brain Bank (REC Reference: 15/SC/0639); and (v) South West Dementia Brain Bank (NRES Committee South West Central Bristol, REC reference: 08/H0106/28 + 5).

### Immunohistochemistry

Immunohistochemistry was performed on 6 μm thick sections, with appropriate antigen retrieval steps. The primary antibodies (Supplementary Table 2) were used to recognize: (i) epitopes of pTau-—AT8, AT100, CP13, PHF1, pThr181, pSer356 and pSer396, and phosphorylated and unphosphorylated tau with Tau-2; (ii) microglial markers^23,24^—Iba1, CD68, HLA-DR, CD64, CD32a and CD16; (iii) astrocyte markers—GFAP, EAAT2, glutamine synthetase (GS), ALDH1L1; and (iv) T lymphocyte markers CD4 and CD8. Incubation with secondary biotinylated antibodies (Vector Laboratories) was visualized by avidin-biotin-peroxidase complex (Vectastain Elite, Vector Laboratories) and chromogenic reaction with 3,3′-diaminobenzidine (Vector Laboratories). Slides were counterstained with haematoxylin and coverslipped with Expert XTF Mounting Media (CellPath). Due to the large number of slides, staining was carried out in three batches. Each batch included all conditions and brain banks. One set of slides was stained with Haematoxylin and Eosin (H&E) to examine the cortical tissue integrity.

### Image acquisition and analysis

Digital images of the whole tissue section were acquired at magnification × 20 with an automated Olympus VS110 slide scanning microscope (Olympus America Inc.) and regional extraction was carried out in the grey matter using the CSG add-on to the Olympus VS-Desktop software.^23,25^ Forty regions of interest (ROIs) (500 × 500 pixels or 172.75 µm × 172.75 µm) were extracted in a zigzag manner along the grey matter. ROI analysis was carried out with Fiji ImageJ v1.53c software (NIH, USA),^26^ which thresholded the ROIs at a pre-selected value and calculated the percentage of the image that was stained with a specific marker, expressed as protein load (%). This was then averaged across all 40 images to obtain a protein load per case.

Microglial cell counts were performed from the ROIs immunolabelled with Iba1. A minimum of 100 cells were categorized by morphology as previously defined^27^ and used by us and others^28-30^: ramified microglia (small cell body with ≥4 thin highly branched processes), reactive microglia (increased cell body size with 2–3 processes with fewer and smaller branches) and amoeboid microglia (large cell body size with 1–2 shortened processes or none).

H&E images were analysed using QuPath v0.2.3 software (The University of Edinburgh, UK).^31^ The ‘Create Thresholder’ function was used to achieve a resolution suitable for differentiating tissue from areas of vacuolation within the cortex. The percentage area of residual tissue reflected the cortical tissue integrity, with lower cortical integrity values corresponding to status spongiosus (i.e. cortical neuropil and neuronal degeneration).^32^

CD4^+^ and CD8^+^ T cells were quantified with QuPath. Due to the low number of cells present in the tissue, the entire section was circumscribed to create an ROI that was free from any imaging artefacts, debris or meninges and specific from the grey matter. Positive staining was detected using the ‘Fast Cell Counts’ function and detected cells were manually categorized by compartment (perivascular or parenchyma).

### Inflammatory protein multiplex assay

Inflammatory proteins were measured on the V-Plex Meso Scale Discovery (MSD) electrochemiluminescence multi-spot assay platform (MesoScale Diagnostics). Frozen samples of grey matter were prepared as per the manufacturer’s protocol. Brain homogenates were used for the V-Plex Chemokine Panel 1 (Eotaxin, Eotaxin-2, TARC, IP10, MIP1α, MIP1β, IL8, MCP1, MDC and MCP4), Cytokine Panel 1 (GM-CSF, IL1α, IL5, IL7, IL12/IL23p40, IL15, IL16, IL17A, TNFβ, VEGF) and Proinflammatory Panel 1 (IFNγ, IL1β, IL2, IL4, IL6, IL8, IL10, IL12p70, IL13, TNFα). Each plate was read on a Meso Quickplex SQ120 with absolute target protein levels (pg/ml) obtained and normalized to the total protein amount.

### Statistical analysis

Statistical analysis was performed using GraphPad Prism v8.4.3 (GraphPad Software, San Diego, USA). Data are presented as combined disease groups (designed FTLD-tau) and separately by disease category. Normality was tested with the Kolmogorov-Smirnov’s test. Comparisons between controls and FTLD-tau cases were performed using *t*-test or Mann-Whitney U-test and between study groups and controls using a one-way ANOVA (and Holm-Sidak’s *post hoc* test) or Kruskal-Wallis test (and Dunn’s *post hoc* test) depending on normality to compare. Correlation between the different markers within each study group was assessed with Pearson’s or Spearman's test depending on normality. To account for multiple testing, the Benjamini, Krieger and Yekutieli procedure to control for the false discovery rate (FDR) was used as *post hoc* correction. Adjusted *P-*values < 0.05 for intergroup comparisons and 0.01 for correlations were considered significant.

## Results

### Tau pathology: qualitative observations

**Figure 1:**
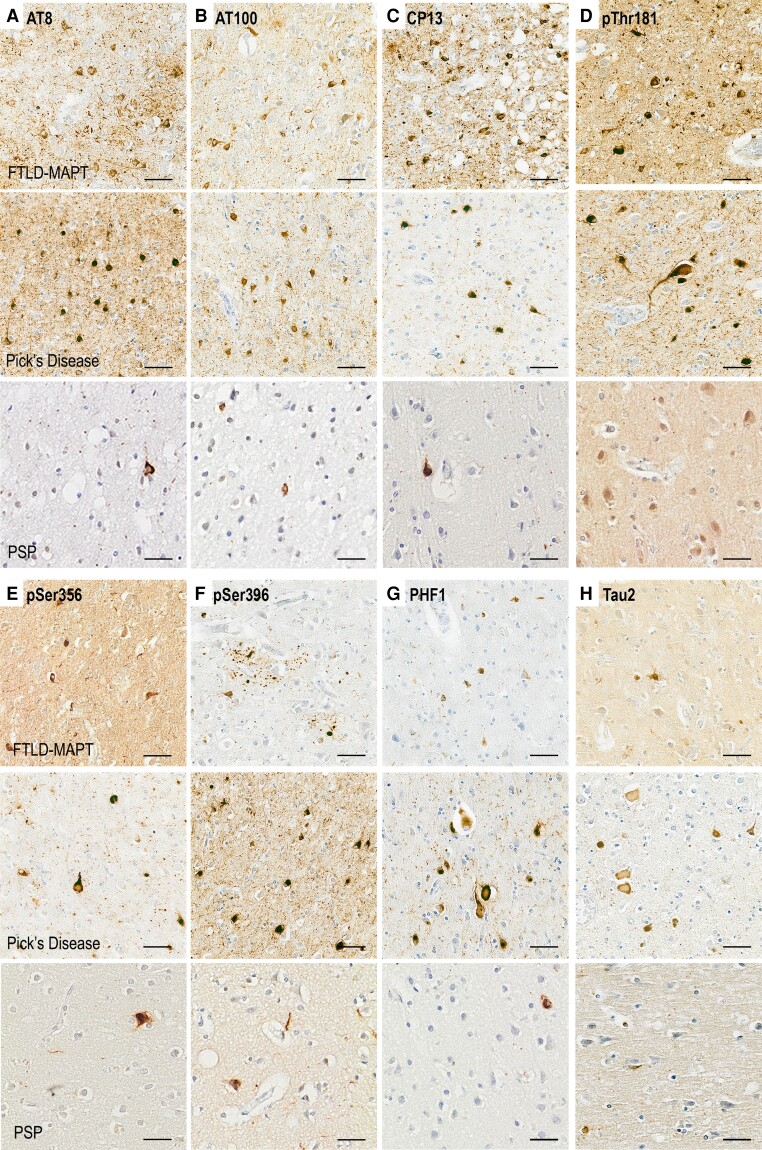
**Illustrations of phosphorylated and unphosphorylated tau staining across FTLD-tau subtypes.** Examples of each staining with haematoxylin counterstaining. (**A**) AT8—tau phosphorylated at Ser202 and Thr205. (**B**) AT100—tau phosphorylated at Thr212 and Ser214. (**C**) CP13—tau phosphorylated at Ser202. (**D**) Thr181—tau phosphorylated at Thr181. (**E**) Ser356—tau phosphorylated at Ser356. (**F**) Ser396—tau phosphorylated at Ser396. (**G**) PHF1—tau phosphorylated at Ser396 and Ser404. (**H**) Tau-2—phosphorylated and unphosphorylated tau. Scale bars = 50 μm. FTLD = frontotemporal lobar degeneration; PSP = progressive supranuclear palsy.

The hallmark signs of FTLD-tau pathology were revealed by the majority of tau antibodies (Fig. 1 and Supplementary Fig. 1). Antibodies AT8, AT100, CP13, pThr181 and pSer396 stained similar features showing Pick bodies, Pick cells and neuropil threads and grains in PiD (Supplementary Fig. 1A–D). Whilst the large pTau neuronal features, such as neurofibrillary degeneration, Pick bodies and Pick cells, were stained by most antibodies, neuropil thread and grains were more pronounced in pThr181 staining (Fig. 1D) compared with pSer356, PHF1 and Tau-2 antibodies (Fig. 1E, G and H).

Among the different disease subtypes, FTLD-MAPT cases showed a moderate amount of pTau neuronal neurofibrillary degeneration along with some neuropil thread and grain staining (as in PiD, Supplementary Fig. 1C and D). There was also staining of glial cells, similar to the ‘tufted astrocytes’, as reported in PSP (Supplementary Fig. 1F).

In contrast, PiD cases showed severe pathology in the grey matter, including Pick bodies and Pick cells/ballooned neurons (Supplementary Fig. 1A and B). The neuropil staining was prevalent with the identification of neuropil threads and grains of pTau (Supplementary Fig. 1C). Some cases had plaque-like features that appear similar to the ‘astrocytic plaques’ (Supplementary Fig. 1E) described in another FTLD-tau disease, corticobasal degeneration.^4^

PSP with FTD cases generally had little cortical tau pathology with few neurofibrillary tangles but relatively abundant glial cell staining (likely tufted astrocytes) (Supplementary Fig. 1F). However, the proportion of neuron to glial tau staining was variable among PSP cases.

### Quantification of tau pathology and cortical tissue integrity

**Figure 2:**
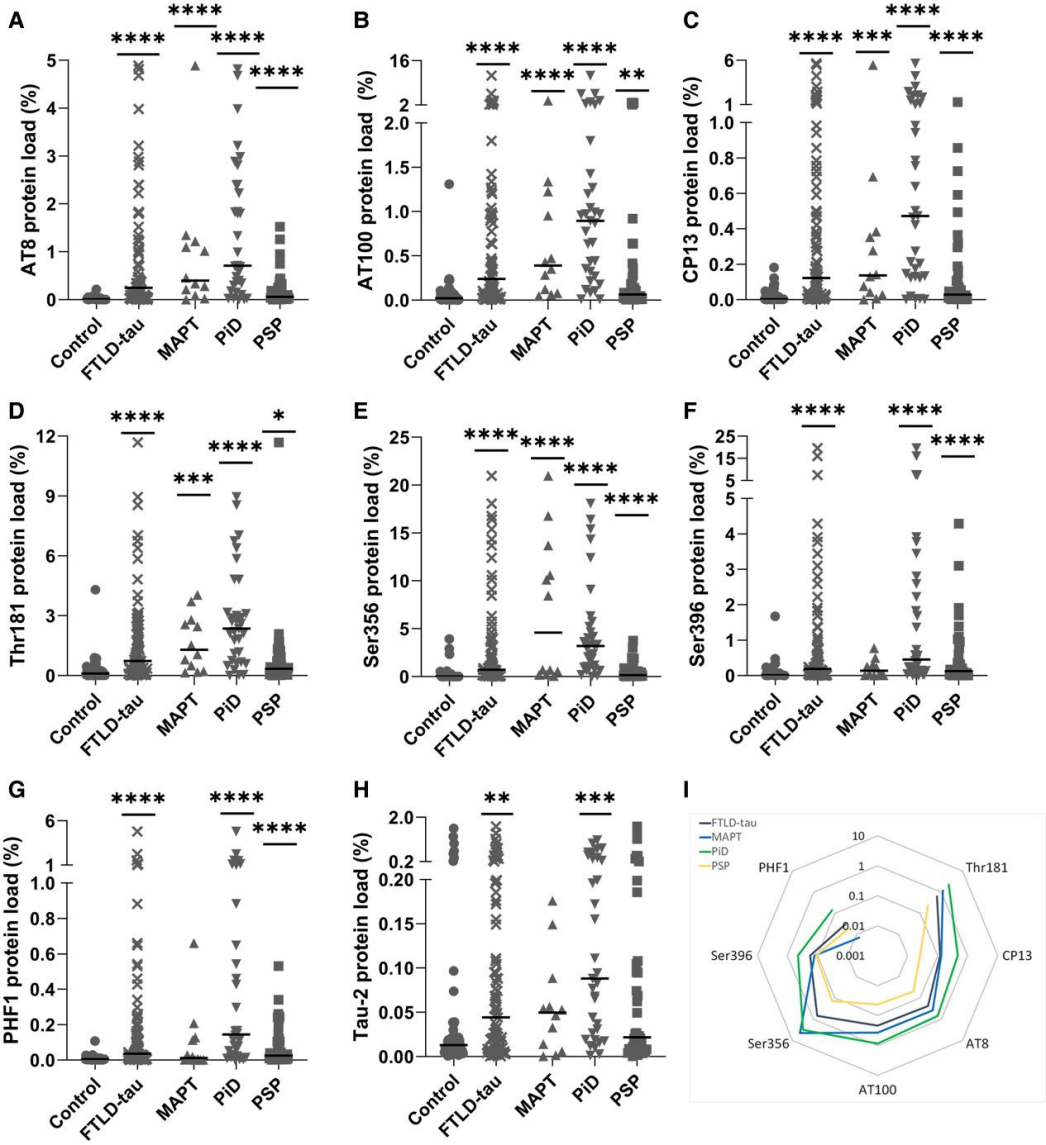
**Quantification of tau across FTLD-tau subtypes**. Protein load (%) presented for controls (*n* = 52), combined FTLD-tau (*n* = 90) and FTLD subtypes: FTLD-MAPT (MAPT, *n* = 12), Pick’s disease (PiD, *n* = 33), and progressive supranuclear palsy (PSP, *n* = 45). Graphs show individual case and median values. (**A**) AT8—tau phosphorylated at Ser202 and Thr205. (**B**) AT100—tau phosphorylated at Thr212 and Ser214. (**C**) CP13—tau phosphorylated at Ser202. (**D**) Thr181—tau phosphorylated at Thr181. (**E**) Ser356—tau phosphorylated at Ser356. (**F**) Ser396—tau phosphorylated at Ser396. (**G**) PHF1—tau phosphorylated at Ser396 and Ser404. (**H**) Tau-2—phosphorylated and unphosphorylated tau. Control versus FTLD-tau and control versus MAPT/PiD/PSP were analysed separately by Mann-Whitney U-test and Kruskal-Wallis test followed by Dunn’s *post hoc* test, respectively. *P < 0.05; **P < 0.01; ***P < 0.001; ****P < 0.0001. (**I**) Radar graph showing tau epitope abundance versus controls from N- to C-terminal domains. The radius of the circle represents the averaged variation from controls. Black = FTLD-tau; blue = MAPT; green = Pick’s disease; yellow = PSP; FTLD = frontotemporal lobar degeneration.

All pTau epitopes showed increased expression in FTLD-tau versus controls (*P <* 0.0001). Several tau hyperphosphorylation epitopes displayed a similar pattern of expression between groups, with significantly increased expression in all disease groups versus controls (Fig. 2A–E): *AT8 (pSer202/pThr205):* FTLD-MAPT cases (*P <* 0.0001), PiD (*P <* 0.0001) and PSP (*P <* 0.0001); *AT100 (pThr212/pSer214):* FTLD-MAPT cases (*P <* 0.0001), PiD (*P <* 0.0001) and PSP (*P =* 0.0045); *CP13 (pSer202):* FTLD-MAPT cases (*P =* 0.0002), PiD (*P <* 0.0001) and PSP (*P <* 0.0001); *pThr181:* FTLD-MAPT cases (*P =* 0.0001), PiD (*P <* 0.0001) and PSP (*P =* 0.0136); and *pSer356:* FTLD-MAPT cases (*P <* 0.0001), PiD (*P <* 0.0001) and PSP (*P <* 0.0001). PSP consistently showed a lower pTau protein load compared to PiD across tau epitopes. Of note, the expression of pSer356 was greatest in FTLD-MAPT cases, whereas the expression of other markers was highest in PiD. Data are presented in the Supplementary Table 3A.

Unlike the epitopes above, pTau epitopes phosphorylated at pSer396 or pSer396/pSer404 (PHF1) were not significantly different between FTLD-MAPT and controls (Fig. 2F and G), while PiD and PSP cases were found to be significantly increased versus controls, (PiD: pSer396 *P <* 0.0001; PHF1 *P <* 0.0001; PSP: pSer396 *P <* 0.0001; PHF1 *P <* 0.0001). The Tau-2 protein load was only increased in PiD versus controls (*P <* 0.0001), while the other disease groups showed no difference (Fig. 2H). This could be explained by the lower specificity of Tau-2 antibody for targeting aggregated tau and thus a staining more likely to be observed in brains with higher pTau expression, such as in PiD cases. All values normalized to controls are presented in a radar plot for comparison (Fig. 2I).

**Figure 3:**
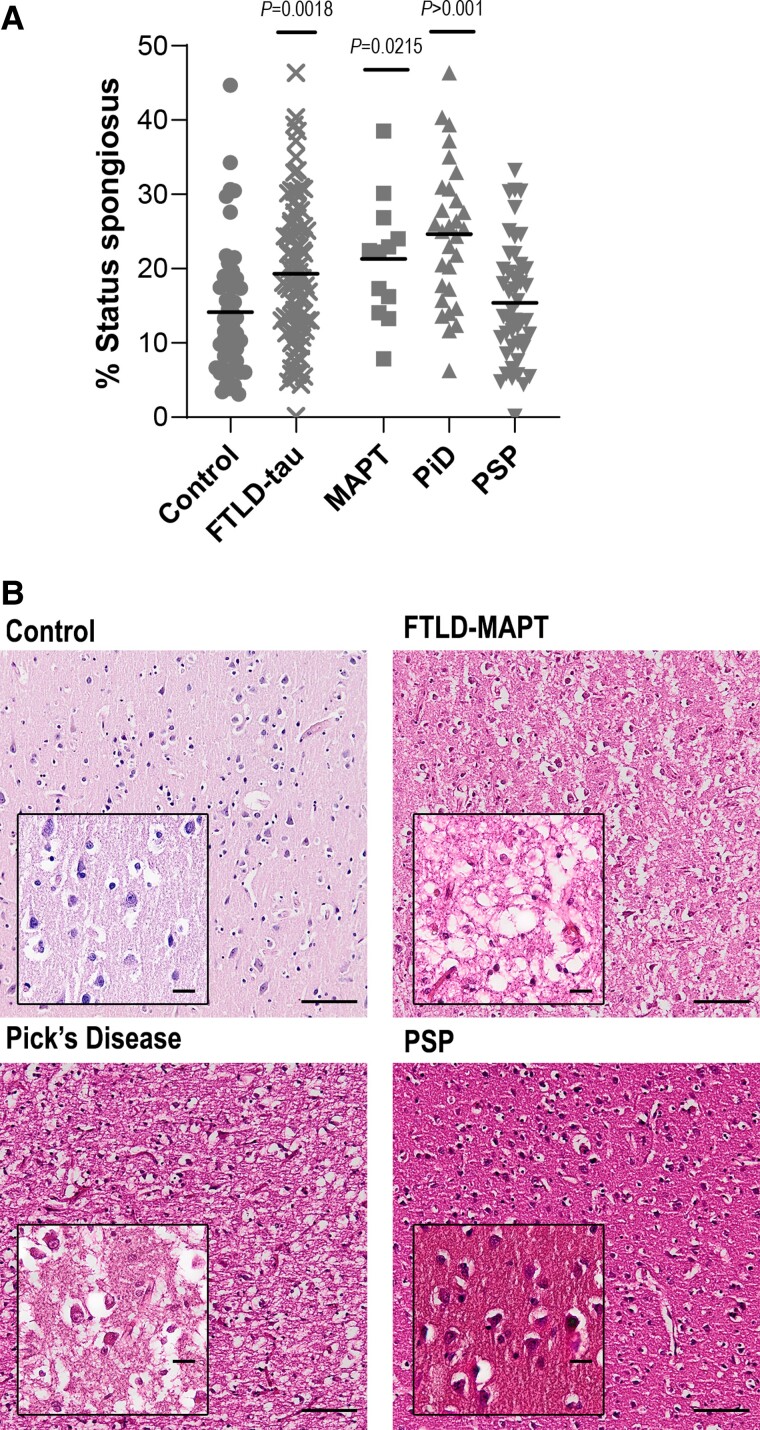
**Cortical neurodegeneration across FTLD-tau subtypes.** (**A**) Cortical neurodegeneration measured as status spongiosus based on haematoxylin and eosin stain in controls (*n* = 51), combined FTLD-tau (*n* = 89) and FTLD subtypes: FTLD-MAPT (MAPT, *n* = 12), Pick’s disease (PiD, *n* = 32) and progressive supranuclear palsy (PSP, *n* = 45). Control versus FTLD-tau and control versus MAPT/PiD/PSP were analysed separately by *t*-test and one-way ANOVA followed by Holm-Sidak’s *post hoc* test, respectively. Graphs show individual points and mean values. (**B**) Illustrations of haematoxylin and eosin stain in control and FTLD-tau subtypes. Scale bar = 100 μm, *Inset* = 20 μm. FTLD = frontotemporal lobar degeneration.

Cortical integrity assessment in FTLD-tau showed reduced neuropil integrity (i.e. increased status spongiosus) versus controls (*P* = 0.0018). More specifically, FTLD-MAPT and PiD cases had significantly reduced tissue integrity (*P =* 0.0215 and *P* > 0.0001, respectively), while PSP cases were not affected (Fig. 3 and Supplementary Table 3A).

### Microglial activation, number and morphology

**Figure 4:**
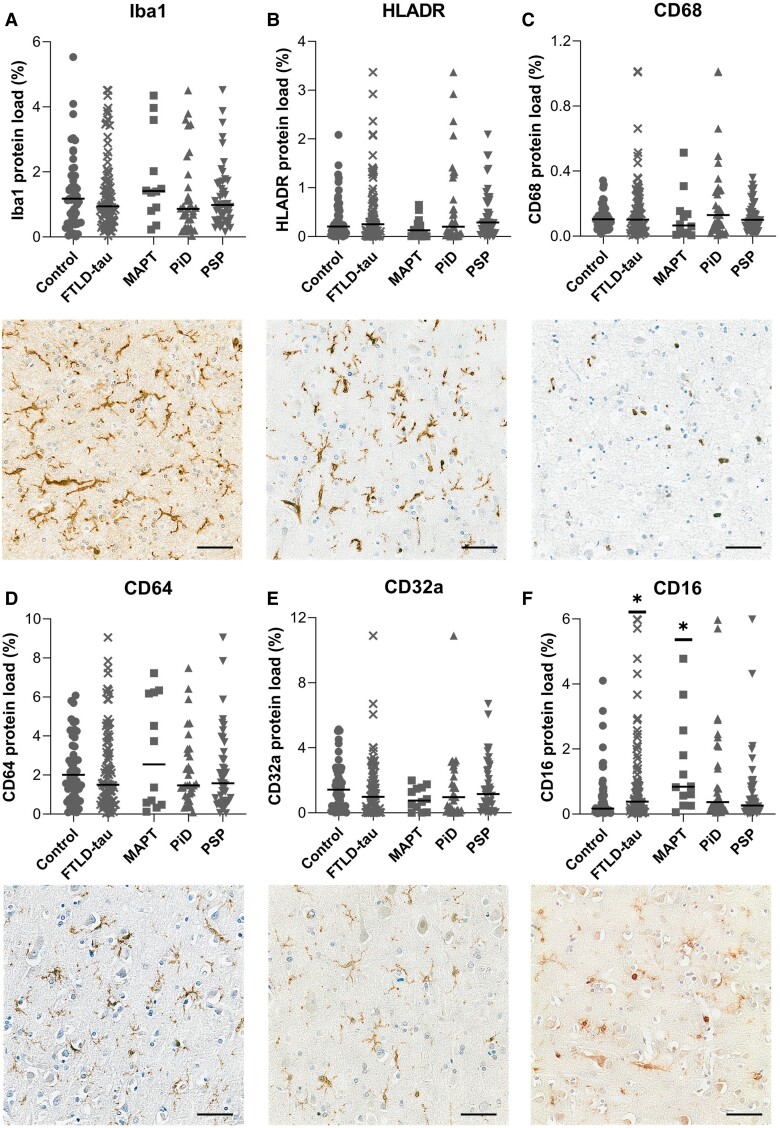
**Quantification and illustration of microglial staining across FTLD-tau subtypes.** Protein load presented (%) for controls (*n* = 52), combined FTLD-tau (*n* = 90) and by FTLD subtypes: FTLD-MAPT (MAPT, *n* = 12), Pick’s disease (PiD, *n* = 33) and progressive supranuclear palsy (PSP, *n* = 45). (**A**) Iba1, (**B**) HLA-DR, (**C**) CD68, (**D**) CD64/FCγRI, (**E**) CD32a/FCγRIIa and (**F**) CD16/FCγRIII. Control versus FTLD-tau and control versus MAPT/PiD/PSP were analysed separately by Mann-Whitney U-test and Kruskal-Wallis test followed by Dunn’s *post hoc* test, respectively. Graphs show individual points and median values. *P < 0.05; **P < 0.01; ***P < 0.001; ****P < 0.0001. Images illustrate the staining for each marker counterstained with haematoxylin. Scale bar = 50 μm. FTLD = frontotemporal lobar degeneration.

Microglial activation in FTLD-tau was assessed with markers related to specific functions, including motility (Iba1),^24,33^ antigen presentation (HLA-DR),^34^ phagocytosis (CD68)^35^ and the Fcγ receptors, key effectors of immunoglobulin activity (CD64, CD32a and CD16),^36^ in order to understand the behaviour of the cells. Some of these markers are also expressed by other immune cells, such as the CD16 found on the surface of natural killer cells, neutrophils, monocytes or T cells. However, in our cohorts, all markers labelled microglia (Fig. 4) and perivascular macrophages only.

Quantification of Iba1, HLA-DR, CD68, CD64 and CD32a loads (Fig. 4A–E) showed no difference between disease groups and controls. However, CD16 was increased in FTLD-tau (*P =* 0.0292) and specifically in FTLD-MAPT cases versus controls (*P =* 0.0150) (Fig. 4F and Supplementary Table 4).

**Figure 5:**
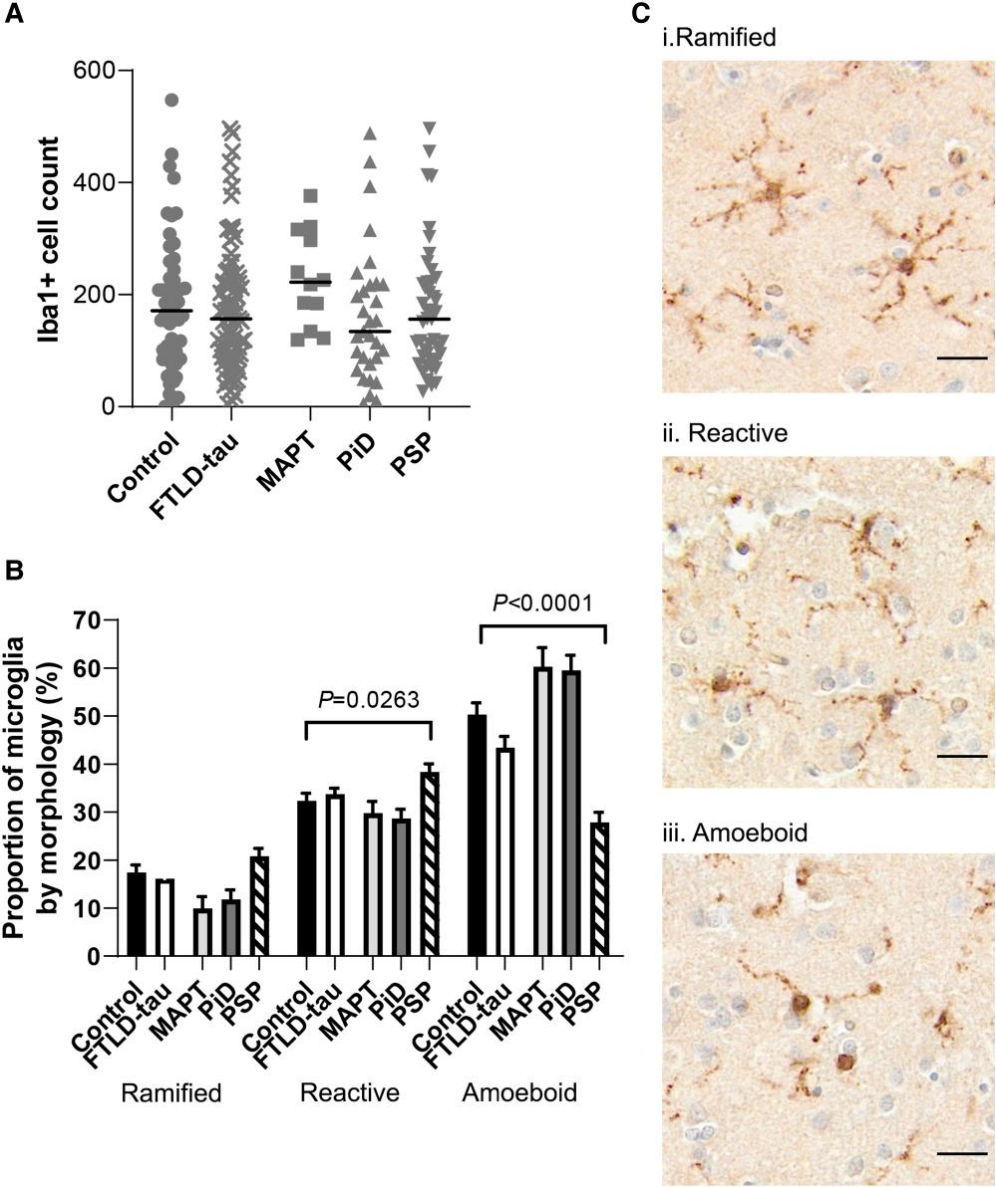
**Quantification of microglial cell number and semiquantitative analysis of morphology.** (**A**) Graph showing Iba1^+^ microglial cell counts between control (*n* = 52) and combined FTLD-tau (*n* = 88) cases as well as for separate subtypes: FTLD-MAPT (MAPT, *n* = 12), Pick’s disease (PiD, *n* = 31) and progressive supranuclear palsy (PSP, *n* = 45). Control versus FTLD-tau and control versus MAPT/PiD/PSP were analysed separately by Mann-Whitney U-test and Kruskal-Wallis test, respectively. Graph shows individual points and median values. (**B**) Graph showing the proportion of Iba1^+^ cells that are ramified, reactive or amoeboid for control (black), combined FTLD-tau (white), MAPT (light grey), PiD (dark grey) and PSP (striped) cases. Control versus FTLD-tau and control versus MAPT/PiD/PSP were analysed separately by Mann-Whitney U-test and Kruskal-Wallis test followed by Dunn’s *post hoc* test, respectively. (**C**) Images illustrating the different microglial morphologies classified as (**i**) ramified, (**ii**) reactive and (**iii**) amoeboid. Scale bar = 50 μm. FTLD = frontotemporal lobar degeneration.

Cell counts showed no difference between disease groups and controls (Fig. 5A). When assessing the proportion of microglial morphologies (Fig. 5B and C), no disease group had a significantly altered proportion of ramified microglia (≥4 processes) versus controls. In PSP, a higher proportion of microglia was identified as reactive (2–3 processes) (PSP *=* 38.3% versus controls = 32.3%, *P =* 0.0263); whilst significantly less were classed as amoeboid microglia (PSP *=* 27.8% versus control = 50.3%, *P <* 0.0001). Compared to controls, PiD and FTLD-MAPT showed a trend towards a reduction in the proportion of ramified cells (controls = 17.4%, FTLD-MAPT = 9.9%, PiD = 11.8%) and an increase in amoeboid cells (controls = 50.3%, FTLD-MAPT = 60.3%, PiD = 59.5%) but these differences were not significant.

Altogether, these data imply that microglial activation is minimal in FTLD-tau, with potential differences in PSP compared to FTLD-MAPT and PiD. Indeed, in PSP, microglia are significantly more ramified and less amoeboid, consistent with an overall reduced cortical pathology.

### Astrocyte reactivity

Tau accumulates in astrocytes and these latter also react to neuronal degeneration, both of which may affect their function. We addressed this by exploring four markers: glial fibrillary acidic protein (GFAP), the major protein constituent of astrocyte intermediate filament, upregulated in reactive astrocytes; aldehyde dehydrogenase-1 L1 (ALDH1L1), a pan-astrocyte cytosolic enzyme; excitatory amino acid transporter 2 (EAAT2/GLT-1), the predominant astrocyte glutamate transporter; and GS, a cytosolic enzyme responsible for the breakdown of glutamate into glutamine.

**Figure 6:**
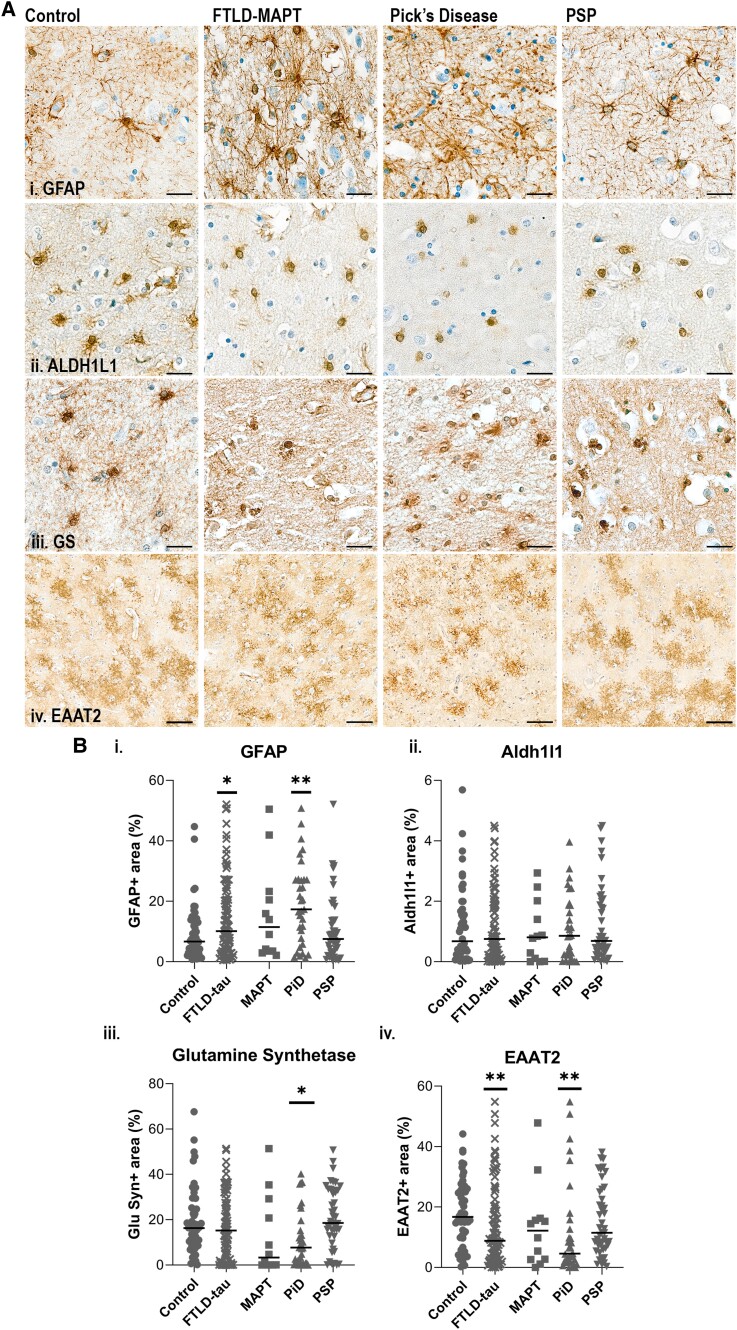
**Illustrations and quantification of astrocyte stains across FTLD-tau subtypes.** Illustrations of each staining with haematoxylin counterstaining. [**A**(**i**)] glial fibrillary acidic protein (GFAP), [**A**(**ii**)] aldehyde dehydrogenase (ALDH1L1), [**A**(**iii**)] glutamine synthetase (GS) and [**A**(**iv**)] EAAT2/GLT-1. GFAP, GS and ALDH1L1: scale bars = 25 μm, EAAT2: scale bar = 100 μm. Protein load (%) of controls (*n* = 52), combined FTLD-tau (*n* = 89) and FTLD subtypes: FTLD-MAPT (MAPT, *n* = 12), Pick’s disease (PiD, *n* = 32) and progressive supranuclear palsy (PSP, *n* = 45). [**B**(**i**)] GFAP, [**B**(**ii**)] ALDH1L1, [**B**(**iii**)] GS and [**B**(**iv**)] EAAT2/GLT-1. Control versus FTLD-tau and control versus MAPT/PiD/PSP were analysed separately by Mann-Whitney U-test and Kruskal-Wallis test followed by Dunn’s *post hoc* test, respectively. Graphs show individual points and median values. *P < 0.05; **P < 0.01; ***P < 0.001; ****P < 0.0001. FTLD = frontotemporal lobar degeneration.

All subtypes of astrocytes were stained with GFAP.^7^ ALDH1L1 and GS antibodies stained only the cell body and local projections, but staining was generally spread out through the grey matter [Fig. 6A(ii) and (iii)]. EAAT2 stained much of the arborization of the astrocytes with the staining appearing ‘fuzzy’ compared to the distinct process stained by GFAP. Whilst the morphology of the EAAT2 + cells was homogenous, the density of staining and location was very variable across groups and between cases in the same group [Fig. 6A(iv)].

Quantification of GFAP showed a significant increase in FTLD-tau overall (*P =* 0.0345) and PiD (*P =* 0.0019) versus controls. There was no difference across disease groups in the expression of pan-astrocytic ALDH1L1. Markers of astrocyte glutamate cycling function were reduced in FTLD-tau versus controls (EAAT2, *P =* 0.0075) and PiD versus controls (EAAT2, *P =* 0.0026; GS, *P =* 0.0387) (Fig. 6B and Supplementary Table 4).

Altogether, these data support astrocyte reactivity in FTLD-tau, associated with decreased glutamate cycling activity of astrocytes.

### Neuroinflammatory profile

**Figure 7:**
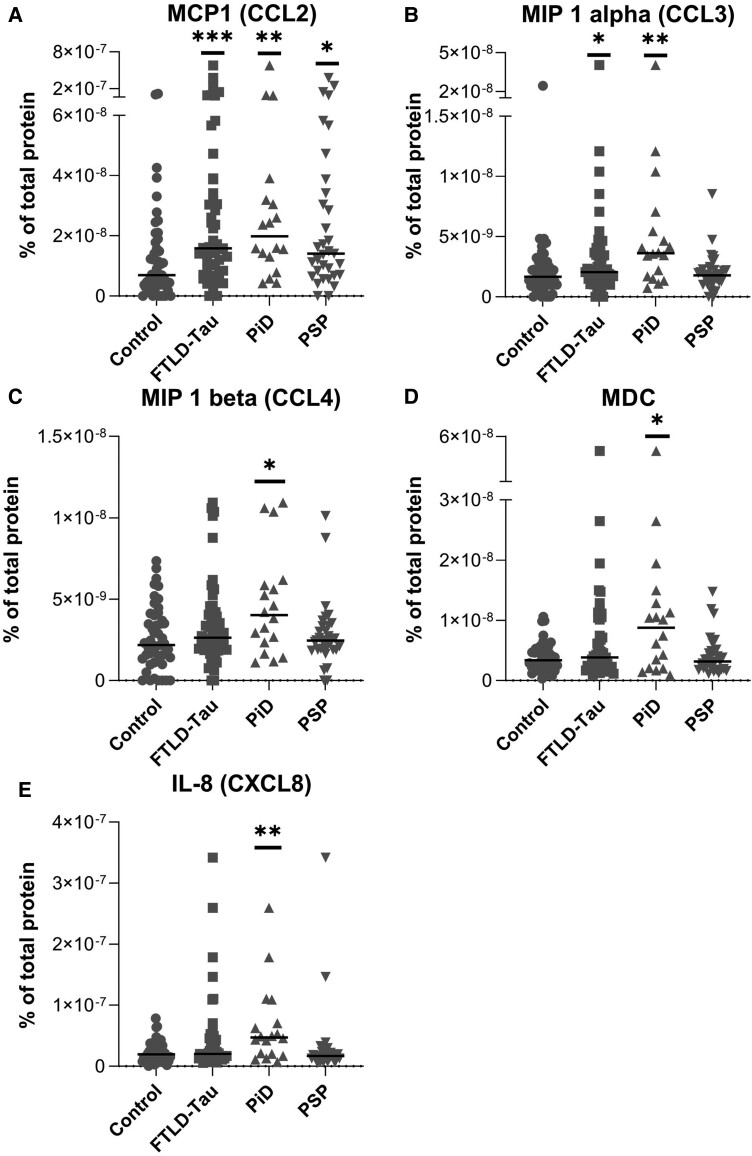
**Quantification of significantly altered chemokine markers from frozen brain tissue.** Graphs show the percentage of the total protein that is composed of the specific analyte, individual cases and median values. Analytes were detected by using the Meso Scale Discovery (MSD) V-Plex Chemokine Panel-1 (human) kit. Controls (*n* = 49), Pick’s disease (PiD, *n* = 18) and progressive supranuclear palsy (PSP, *n* = 33). (**A**) MCP1/CCL2, (**B**) MIP1α/CCL3, (**C**) MIP1β/CCL4, (**D**) IL8/CXCL8 and (**E**) macrophage-derived chemokine (MDC). Analysis was carried out by Mann-Whitney U-test for control versus FTLD-tau and by Kruskal-Wallis tests followed by Dunn’s *post hoc* test for control versus MAPT/PiD/PSP. Graphs show individual points and median values. Note: some graphs have split *y-*axes. *P < 0.05; **P < 0.01; ***P < 0.001; ****P < 0.0001. Analytes that were not significantly different are shown in Supplementary Fig. 4.

To assess the neuroinflammatory environment in the brains of FTLD-tau patients, we measured 30 cytokines, chemokines and proinflammatory factors. From the chemokine panel, two markers showed a difference between FTLD-tau and controls (MCP1/CCL2, *P* = 0.0005; MIP1α/CCL3, *P* = 0.0437). When looking at individual FTLD-tau diseases, five chemokines were significantly upregulated in PiD cases compared to controls: MCP1/CCL2 (*P =* 0.0058), MIP1α/CCL3 (*P =* 0.0023), MIP1β/CCL4 (*P =* 0.0290), IL-8/CXCL8 (*P =* 0.0077) and MDC (*P* = 0.0304) (Fig. 7). The expression of the other chemokines was not altered (Supplementary Fig. 4). None of the analytes on the cytokine panel (Supplementary Fig. 2) and proinflammatory panel were significantly different (Supplementary Fig. 3).

### T lymphocytes

**Figure 8:**
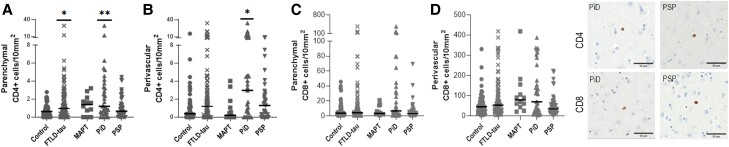
**Parenchymal and perivascular T lymphocytes across FTLD-tau subtypes.** Illustrations and quantification of the (**A**) parenchymal CD4^+^ T cells, (**B**) perivascular CD4^+^ T cells, (**C**) parenchymal CD8^+^ T cells and (**D**) perivascular CD8^+^ T cells for control (*n* = 52), combined FTLD-tau (*n* = 90), FTLD-MAPT (MAPT, *n* = 12), Pick’s disease (PiD, *n* = 33) and progressive supranuclear palsy (PSP, *n* = 45) cases. Analysis was carried out by Mann-Whitney U-test for control versus FTLD-tau and by Kruskal-Wallis tests followed by Dunn’s *post hoc* test for control versus MAPT/PiD/PSP. Graphs show individual cases and median values. *P < 0.05; **P < 0.01. FTLD = frontotemporal lobar degeneration.

We assessed the infiltration of T lymphocytes by quantifying both CD4^+^ and CD8^+^ cells in the grey matter parenchyma and perivascular areas (Supplementary Table 5). Overall, there were few CD4 + lymphocytes but parenchymal CD4^+^ cells were significantly increased in FTLD-tau overall (*P* = 0.0109), with increased CD4^+^ T cells only detected in PiD versus controls in the parenchyma (*P =* 0.0040) and the perivascular space (*P =* 0.0158) (Fig. 8). In combined or individual FTLD-tau disorders, no difference in CD8^+^ cells was observed (Fig. 8). Of note, the overall number of CD8^+^ was much higher than CD4^+^ cells, with a predominance in the perivascular spaces (Supplementary Table 5).

### Correlations between the markers

Analyses were performed within each study group to assess whether the presence of pTau epitopes was correlated with changes in innate or adaptive immune markers and whether there was any association between T-cell infiltration and microglia and/or astrocyte activation.

#### Tau and microglia

In FTLD-tau cases, significant correlations were detected between pTau and HLA-DR, CD68 and CD64 (apart from pThr181 and pSer356). Across the disease groups, CD68 has the most numerous significant associations with pTau followed by HLA-DR and CD64. In controls, significant correlations were observed mostly for CD68 with pTau, with microglial phagocytosis possibly reflecting cellular degeneration associated with the ageing process or early pathology in this population (Supplementary Fig. 5). Of note, no association was detected between pTau and Iba1, the homeostatic microglial marker. Taken together, these data support association between pTau epitopes and microglial activation markers in FTLD-tau.

#### Tau and astrocytes

In FTLD-tau, all tau markers, except pSer356 and pThr181, correlated with GFAP. FTLD-MAPT showed negative associations between GS and both pThr181 and pSer356. In PiD, correlations were observed between GFAP and Tau-2, pSer396 and PHF1, as well as between GS and Tau-2. Similarly, PSP showed associations between GFAP and CP13, pSer396 and PHF1; and between EAAT2 and Tau-2. No association was detected with ALDH1L1. In controls, GFAP significantly correlated with PHF1 and EAAT2 with CP13, consistent with early pathology (Supplementary Fig. 6). These data support that most pTau epitopes positively correlate with the astrocytic reactivity marker GFAP in FTLD-Tau. In parallel, some epitopes inversely correlate with an astrocytic marker of glutamate cycling activity, particularly in FTLD-MAPT.

#### Tau and T lymphocytes

All pTau markers positively correlated with CD8^+^ T cells in the combined FTLD-tau group (Supplementary Fig. 7). Analysis within each FTLD-tau subgroup revealed no associations in FTLD-MAPT. However, CD8^+^ T lymphocytes were correlated in PiD with Tau-2 and PHF1, and in PSP with Tau-2, AT8, CP13, pSer396 and PHF1. There were also significant correlations in the combined FTLD-tau group between CD8^+^ T cells and microglial markers CD68 and CD64, as well as with the astrocyte reactivity marker GFAP. No correlations were found between CD4^+^ T cells and pTau or glial markers (Supplementary Fig. 8). These data support that infiltration of CD8^+^ but not CD4^+^ T cells positively correlates with pTau epitopes as well as with markers of astrocyte reactivity and/or microglial activation in FTLD-tau.

Of note in FTLD-tau, CD4^+^ T cell infiltration was associated with MIP1α/CCL3 (ρ = 0.400, *P* = 0.004), IL15 (ρ = 0.380, *P* = 0.006) and IL8 (ρ = 0.400, *P* = 0.004), and CD8^+^ T cells with MIP1α/CCL3 (ρ = 0.360, *P* = 0.009).

## Discussion

This study is the first to assess several microglial and astrocytic markers in a large cohort of FTLD-tau cases. Previous publications presented case reports, semi-quantitative data or smaller cohorts.^7^ Here, we observed in FTLD-tau that: (i) microglia did not seem phenotypically active but are associated with the presence of pTau epitopes; (ii) astrocytes are reactive and may be functionally altered, especially with regards to glutamate cycling; and (iii) the presence of T lymphocyte infiltration, mainly in PiD, associated with expression of chemokines/cytokines related to T cell recruitment and/or survival, particularly MIP1α/CCL3 and IL15, respectively.

We acknowledge some limitations when interpreting our results. First, our investigation is limited to the end stage of disease and thus was unable to provide insight into the course of the disease. Effort was made to select cases with controlled parameters (e.g. post-mortem delay) for optimal detection, with cases matched as closely as possible to controls from the same brain bank. There was variability in the cohorts, which may be due to interindividual genetic variability or other factors affecting the individuals during their lifetime, such as infection, chronic comorbidities, etc. We also acknowledge that PSP primarily affects the basal ganglia and brainstem,^5,37,38^ with the frontal cortex being a secondarily affected area. However, our study assessed cases that had a neuropathological diagnosis of PSP with FTD, therefore requiring the presence of cerebral cortical pathology. These conditions often present an asymmetric neuropathology, thus limiting the comparison between the protein expression patterns and immune cells, with the multiplex assays performed in the same anatomical area but in different hemispheres.

### Pathological tau features and cortical tissue integrity

As expected, tau pathology was increased in all subtypes of FTLD-tau compared to controls. Of note, the Tau-2 antibody recognizes both phosphorylated and non-phosphorylated forms of tau but the sum of the staining with the antibodies that recognize specific forms of pTau did not equal the Tau-2 staining. This may relate to differences in antibody binding affinity and/or other methodological considerations. However, each disease showed different profiles of tau phosphorylation relating to the location of the phosphorylation sites on the tau protein. AT8 (pSer202/pThr205), AT100 (pThr212/pSer214), CP13 (pSer202) and pThr181 had similarities in their expression across diseases. This could be explained by their phosphorylation loci being in the proline-rich domain of the tau protein structure.^39^ Therefore, the presence of tau phosphorylated in the proline-rich region appears to be a feature of FTLD-tau but may not be disease subtype-specific although the abundance was highest in PiD. The pSer356 phosphorylation site, in the microtubule-binding domain of tau, was suggested to reflect an early pathological event in tau phosphorylation and aggregation.^40^ Its expression compared to controls was the highest of all tau epitopes, with the greatest in FTLD-MAPT and lower in PiD > PSP, in contrast to the pattern of proline-rich region epitopes. Interestingly, pSer396 and PHF1 (pSer396/pSer404) expression, both with the phosphorylation sites in the c-terminal domain of the tau protein, was modest but supports further evidence of mechanistic differences between FTLD-MAPT and other FTLD-tau diseases, with FTLD-MAPT showing no significant difference in abundance of either epitope. Whilst the cause of the reduced pSer396/p404 signal is uncertain, the pattern of epitopes phosphorylated in FTLD-MAPT is distinct from PiD and PSP. This is interesting considering that FTLD-MAPT is a genetically determined form of FTLD-tau, unlike the sporadic conditions of PiD and PSP.^41,42^ The differences in the patterns of pTau expression is illustrated in Supplementary Table 3B.

FTLD-tau as a group had severe cortical neurodegeneration, which was prominent in FTLD-MAPT and PiD but not in PSP. Our observation is consistent with previous post-mortem reports of severe grey matter atrophy in the frontal lobe in patients with PiD^43,44^ or MAPT mutations^45^ and a study of FTLD-tau patients who had MRI scans followed by post-mortem examination demonstrated that the cortical atrophy was associated with tau pathology. Study of PSP has led to conflicting results, with some studies showing a lack of atrophy in the prefrontal cortex,^46,47^ whilst others confirmed its presence.^48-50^ Our study used cases that were neuropathologically diagnosed as PSP with clinical FTD but nevertheless had relatively little tau pathology or cerebral cortical degeneration. Thus, the difference in the severity of cortical pathology could be explained by the subcortical pathology in PSP leading to death before the pathology in the frontal cortex reached the severity seen in PiD.

#### Microglial immunophenotypic markers

Contrary to other studies into tauopathies, mainly driven by experimental models, microglia did not appear to be strongly activated in FTLD-tau, only showing increased expression of CD16 in FTLD-MAPT. CD16/FcγRIII is a low-affinity immunoglobulin G receptor expressed by microglia, perivascular macrophages and monocytes.^23^ It is unclear whether CD16 expression in FTLD-MAPT was due to microglial expression and/or recruitment of monocytes/macrophages. However, FTLD-MAPT findings support a different underlying mechanism of this condition, with potentially a differential role for microglia in response to tau mutations (and thus altered neuronal function) compared to sporadic tau accumulation. Previous post-mortem FTLD-tau studies reported evidence of microglial activation in combined subtypes of FTLD-tau using semi-quantitative analyses^16^ or smaller cohorts of PiD (CD68, *n* = 6)^51^ or PSP cases (HLA-DR, *n =* 5).^52^ One recent post-mortem study investigating microglia in a range of FTLD, reported absence of CD68 and Iba1 expression in PSP and FTLD-MAPT but increased expression in PiD,^15^ in contrast to our findings, perhaps explained by the limited number of FTD-tau cases (*n* = 5) in that study. Nevertheless, these findings are consistent with a lack of overt microglial activation in FTLD-tau.

Since our study captures FTLD-tau at the end stage of the disease, we cannot conclude whether microglia may play a role earlier in the disease progression, as implied by the PET scan studies using the microglial TSPO ligands ^11^C-PK11195^53^ and ^11^C-PBR28.^54^ It must be noted that without neuropathological examination, these studies grouped participants by clinical syndrome rather than confirmed neuropathological diagnosis. However, some studies have shown increased ^11^C-PK11195 binding in subcortical regions of clinically diagnosed PSP patients^55^ as well as in the frontal lobe.^56^ A study combining PET imaging for microglia and tau detected an association between neuroinflammation (^11^C-PK-11195) and tau deposition (^18^F-AV-1451) in FTD.^57^ Being an *in vivo* study, the patients were not stratified by the subtype of the FTLD (e.g. tau, TDP43). However, the PET findings were reinforced with a post-mortem examination in three cases, confirming the association between microglial density and tau pathology. Another study that monitored patients with genetically confirmed *MAPT* mutations found an overlap between increased tau deposition (^18^F-AV-1451) and neuroinflammation (^11^C-PK-11195),^58^ supporting a link between tau pathology and neuroinflammation during the course of the disease. Consequently, looking at earlier disease stages in humans by PET imaging,^18^ microglial activation may be one of the earliest features of FTLD. Our study highlighted a relationship between pTau epitopes and microglia was present at the end stage of the diseases. Indeed, despite microglia being not phenotypically activated, associations were detected in PiD between microglial markers (CD68, HLA-DR and CD64) and pTau markers (AT00, PHF1, Tau-2 and Ser396) still supporting a link between tau pathology and microglial activation. Our observation was consistent with the study in PiD patients, which showed that the density of pathology (measured by counts of glial inclusions and Pick bodies) was associated with CD68^+^ and amoeboid microglia.^18^

Whilst the alterations in microglial immunophenotypic markers and Iba1^+^ cell counts were limited, changes were noted in microglial morphology across FTLD-tau subtypes. PSP was associated with less amoeboid microglia and more reactive/ramified cells, conversely PiD and FTLD-MAPT showed trends towards more amoeboid and less reactive/ramified cells. Hence, the cell morphology supported a trend towards an activated phenotype in PiD/FTLD-MAPT, which was not corroborated by the immunophenotypic markers, but remain in line with our correlation analyses.

Overall, we did not observe overt microglial activation in FTLD-tau, which may have different neuroinflammatory characteristics than other chronic neurodegenerative diseases associated with tau deposition such as Alzheimer’s disease.^23,59^ We cannot exclude that at the time of the examination, earlier activation might have resolved, or become impaired or exhausted.^23^ Nevertheless, interactions between microglia and pTau emphasize a link between them, with FTLD-MAPT potentially having a differential microglial reactivity pattern.

#### Astrocytes

Changes in the expression of astrocytic immunophenotypic markers were observed in PiD with presence of increased reactive GFAP^+^ astrocytosis^10^ in FTLD-tau overall and specifically in PiD, consistent with a previous study in a smaller cohort.^60^ Increased GFAP has been suggested to indicate astrocytic phenotypic changes rather than astrocyte proliferation,^61^ which is supported in our data by unchanged expression of ALDH1L1, a constitutive pan-astrocyte marker.^62^

During the course of the disease, CSF levels of GFAP are thought to represent a readout of astrocyte reactivity. In an FTLD-MAPT study, GFAP levels were not different from controls,^63^ but YKL40, another primarily astrocytic marker, was increased in the CSF of FTLD-MAPT patients.^64^ In our study, the relationship between GFAP expression and pTau epitopes suggests a link between tau pathology and astrocyte reactivity.

The increased GFAP in PiD coincided with alterations in astrocytic proteins, which govern the cycling of glutamate from the synapse. EAAT2/GLT-1, a membrane-bound transporter that carries glutamate out of the synaptic cleft, was reduced in PiD implying a reduced capacity for astrocytes to remove glutamate from the synapses. This coincided with a decrease in the enzyme GS, responsible for catalysing the condensation of glutamate to glutamine. This may point to altered astrocyte function as a key player in neurodegeneration, as excess glutamate leads to neuro-excitotoxicity. EAAT2 changes have been reported in tauopathy mice,^65^ with a decrease in EAAT2 and GS in P301S mice, suggesting a commonality between tau pathology and altered astrocyte function.^66^ pTau accumulation in astrocytes may drive their dysfunction as reported in P301L mice, a model of tau pathology expressed in astrocytes.^67^ The presence of pTau^+^ astrocytes in PiD supports a similar process.^68^ However, the reductions in EAAT2 and GS may also be a consequence of neurodegeneration rather than a contributing factor, as there are fewer neurons to carry out glutamatergic synaptic signalling and thus astrocytes may downregulate EAAT2 from their membranes in a need-dependent manner.

#### T lymphocytes

We confirmed the presence of CD4^+^ and CD8^+^ T cells in brain parenchyma across all disease groups, as well as in controls. The presence of T lymphocytes in the healthy human brain has been recently reported unchanged across age^69^ and to similar cortical levels as in patients with various neurological disorders when compared with old controls.^20,70^ Of note, CD8+ T cells were largely predominant versus CD4+ T cells,^20,70^ with most of the lymphocytes in the perivascular spaces,^69^ as in our study. The presence of T cells in aged healthy brains remains unclear, as whether this is part of the regular immune surveillance, explaining the steady cell density or the consequence of ageing, as suggested by preclinical studies, due to systemic immune activation altering the peripheral immune homeostasis^71,72^ or a defective blood–brain barrier.^73^

Our findings highlight enhanced recruitment of CD4^+^ T cells to the parenchyma in FTLD-tau, with more infiltrating cells in PiD. Interestingly, although no significant difference was observed in the infiltration of CD8^+^ T cells, parenchymal and perivascular CD8^+^ largely outnumbered CD4^+^ T cells and were the ones mainly detected in THY-Tau22 mice and in the cortex of three human FTLD-MAPT cases with P310L mutations.^14^ Of note, T-lymphocyte infiltration in the human brain has been reported in Alzheimer’s disease^20,23,74,75^ and their numbers correlated with tau pathology rather than amyloid-β deposition,^70,76^ supporting a link between tau pathology and T cell infiltration. Our observations are also in line with the alteration of the blood–brain barrier reported in neurodegenerative diseases, including PiD.^43,44^

Importantly, the presence of CD8^+^ T cells, but not CD4+ T cells, in the combined FTLD-tau group was correlated with all pTau markers. This suggests that signals underlying T lymphocyte recruitment in tauopathies might be different for CD4^+^ versus CD8^+^ T cells. Whereas increased numbers of CD4^+^ T cells might relate to their unselective recruitment, e.g. as a bystander effect, infiltration of CD8^+^ T cells may rely on the presence and/or the extent of given pTau species. In addition, the correlation between CD8^+^ T cell infiltration and CD68/CD64, as well as GFAP, further suggests the involvement of microglia and astrocytes in the recruitment of CD8^+^ T cells, possibly in response to pTau species. Further studies are needed to better characterize the antigen specificity and functional profile of such brain-infiltrating T cells associated with tauopathies and the underlying mechanisms of their recruitment across brain borders.

### The inflammatory brain environment

Our cytokine/chemokine investigation in the FTLD-tau cohorts found five chemokines increased in PiD: MCP1/CCL2, MIP1α/CCL3, MIP1β/CCL4, IL8/CXCL8 and MDC/CCL22. Of these, MCP1/CCL2 is a major monocyte and lymphocyte chemoattractant,^77-80^ which was proposed to induce T lymphocyte infiltration into the brain.^81^ MIP1α/CCL3 is also chemotactic to T cells^82^ and implicated in brain T lymphocyte recruitment in multiple sclerosis.^83^ MIP1β/CCL4 is involved in T cell adhesion to endothelial cells^84^ and promoted their trans-endothelial migration.^85^ CCR5, the chemokine receptor for CCL3/CCL4, is expressed on the cerebral vascular endothelial cells and leads to the opening of the tight junctions and the transmigration of T lymphocytes into the brain.^86^ Furthermore, IL8 is released primarily by microglia^87^ and can act upon the endothelial cells of the blood–brain barrier to allow cell passage. In rats, the inhibition of CXCR2, the IL8 receptor, blocked T lymphocyte infiltration^88^ and recent report in a mouse model of autoimmune neuroinflammation evidenced that astrocytes attract CXCR2-expressing CD4^+^ T cells to grey matter via chemokine production,^89^ supporting that IL-8 might impact T cell infiltration either in a direct or indirect manner, to be further defined. MDC/CCL22 acts on T cells by upregulating the CCR4 receptor expressed by T lymphocytes, increasing their capabilities to migrate through the blood–brain barrier.^90^ Many of these chemokines are also expressed by the T lymphocytes themselves.^91^ Hence, activated CD4^+^ T cells release CCL3 and CCL4 to promote further chemotaxis of distant CD8^+^ T cells.^92^ Furthermore, lymphocytes close to the signalling source lose their chemotactic bias, possibly due to above-threshold chemokine concentrations.^93^ If this process occurs in humans, it means that infiltrating CD4^+^ and CD8^+^ T lymphocytes might recruit further T cells to the brain. Although CCL3 has also been proven to stimulate myeloid cell chemotaxis,^82^ macrophages did not infiltrate the brain in the THY-Tau22 mice^14^ and we did not observe evidence of macrophage recruitment. In the same THY-Tau22 model, MIP1α/CCL3 and MIP1β/CCL4 (along with RANTES/CCL5) were previously reported to increase and be associated with hippocampal infiltration of CD8^+^ T cells.^14^

The increase in chemokines promoting T cell migration and extravasation along with the enhanced infiltration of CD4^+^ T cells into the brain parenchyma in FTLD-tau and mostly in PiD (that has brain atrophy and abnormalities in brain vasculature^43,44^), as well as the association between CD8^+^ T cell infiltration and pTau epitopes, altogether highlight a previously underestimated feature of the disease. Our study also reveals a potential different pathomechanism between the sporadic PiD with a possible or prominent role for both innate and adaptive immunity in the pathogenesis versus the genetic disorder FTLD-MAPT with a different known aetiology origin.

Our study shows that whilst FTLD-tau disease involves the aggregation of tau, the nature of tau pathology is markedly different regarding the neuropathological features, the location of pathology and phosphorylation characteristics of the tau protein. PiD showed the most severe tau pathology and neurodegeneration, which may translate into heightened and/or altered neuroinflammation in the brain. This inflammation (at least at the end stage of the disease) was mainly driven by loss of homeostasis and reactivity of astrocytes. Involvement of T lymphocytes was supported by their recruitment to the brain parenchyma, with CD8^+^ T cells particularly correlated with the severity of tau pathology. This is likely driven by the cerebral release of chemokines that are both chemoattractant for T cells and might also act on the blood–brain barrier permeability. The potential involvement of microglia was unclear and remains to be further explored.

## Supplementary Material

awad309_Supplementary_Data

## Data Availability

The data that support the findings of this study are available from the corresponding author, upon reasonable request.
