# Supplementary material for: Glial reactivity and T cell infiltration in frontotemporal lobar degeneration with tau pathology
Source: Brain. 2023 Sep 13;147(2):590–606. doi: 10.1093/brain/awad309 (PMC10834257; doi:10.1093/brain/awad309)
Supplement: awad309_Supplementary_Data [file awad309_supplementary_data.pdf]

Glial reactivity and T cell infiltration in Frontotemporal lobar degeneration with tau pathology

Iain J Hartnell[1], Declan Woodhouse[1], William Jasper[1], Luke Mason[1], Pavan Marwaha[1], Manon Graffeuil[1], Laurie C Lau[2], Jeanette L. Norman[3], David S Chatelet[4], Luc Buee[5,6], James AR Nicoll[1,7], David Blum[5,6], Guillaume Dorothee[1,8], Delphine Boche[1]

Authors Affiliations

[1]Clinical and Experimental Sciences, Faculty of Medicine, University of Southampton, Southampton, UK

[2]Clinical and Experimental Sciences, Faculty of Medicine, Sir Henry Wellcome Laboratories, University of Southampton, Southampton, UK

[3]Histochemistry Research Unit, Clinical and Experimental Sciences, Faculty of Medicine University of Southampton, Southampton

[4]Biomedical Imaging Unit, University Hospital Southampton NHS Trust, Southampton, UK

[5]University of Lille, Inserm, CHU Lille, UMR-S1172 - Lille Neurosciences & Cognition, Lille, France

[6]Alzheimer & Tauopathies, LabEX DISTALZ, France

[7]Department of Cellular Pathology, University Hospital Southampton NHS Trust, Southampton, UK

[8]Sorbonne Université, Inserm, Centre de Recherche Saint-Antoine, CRSA, Immune System and Neuroinflammation Laboratory, Hôpital Saint-Antoine, France

Supplementary Table 1. Characteristics of the cohorts based on the primary neuropathological diagnosis

	Cases	Cases with frozen tissue	Gender	Age at death (years)	Duration of illness (years)
Control	52	51	22F:30M	77 (59-95)	n/a
FTLD-MAPT	12	none	7F:5M	61 (53-70)	9 (1-15)
PiD	33	19	11F:22M	69 (56-84)	10 (6-23)
PSP with FTD	45	33	17F28M	75 (53-93)	7 (2-17)

FTLD-MAPT = frontotemporal lobar degeneration with tau mutation in exon 10 + 16; PiD: Pick's disease; PSP = Progressive Supranuclear Palsy; FTD = clinical frontotemporal dementia

Supplementary Table 2. Characteristics of the primary antibodies.

	Antibody	Company/Cat. #	Dilution	Target/Function
**Tau**	Tau-2	Sigma (T5530)	1:5000	Reacts with 55-62 kDa tau in the phosphorylated and non-phosphorylated forms of Tau
	AT8	ThermoFisher (MN1020)	1:500	Phosphorylation sites at Ser202 + Thr205
	AT100	ThermoFisher (MN1060)	1:500	Phosphorylation sites at Thr212 + Ser214
	PHF1	Peter Davies	1:1000	Phosphorylation sites at Ser396 + Ser404
	CP13	Peter Davies	1:1000	Phosphorylation site at Ser202
	pThr181	Abcam (ab75679)	1:1000	Phosphorylation site at Thr181
	pSer356	Abcam (ab51036)	1:500	Phosphorylation site at Ser356
	pSer396	Abcam (Ab109390)	1:1000	Phosphorylation site at Ser396
**Microglia**	Iba1	Wako (019-19741)	1:500	Cytoplasmic protein involved in cytoskeletal reorganization, membrane ruffling of the microglial processes and actin cross-linking needed for cell migration32[33], reflecting microglial motility and migration properties[24].
	HLA-DP, DQ, DR	Dako (M0775)	1:200	HLA-DR is a Major Histocompatibility Class (MHC) II cell surface receptor which presents antigens to cells of the immune system eliciting an immune response, involved in the non-self recognition and upregulated in inflammation[34].
	CD68	Dako (M0876)	1:500	CD68 labels lysosomal and endosomal transmembrane glycoprotein of microglia, indicating phagocytic activity[35].
	CD64 (FcγRI)	R&D Biosystems (AF1257)	1:100	Fcγ receptors are central effectors of immunoglobulins (IgG) mediated immune response[36].
	CD32a (FcγRIIa)	NOVUS (nbp1-84589)	1:200	CD64 has high affinity for the Fc portion of IgG, triggering a monocyte/macrophage response[94]. CD64 expression reflects the

	CD16 (FcγRIII)	R&D Biosystems (AF1597)	1:500	presence of IgG in the brain and thus the involvement of systemic immunity[23,95]. CD32a and CD16 are low/medium affinity receptors for immune complex[96].
**Astrocytes**	GFAP	Dako (GA524)	RTU	Glial fibrillary acidic protein (GFAP) is an intermediate filament and the major component of the astrocyte skeleton[97].
	EAAT2	Abcam (ab41621)	1:5000	Excitatory amino acid transporter (EEAT)-2 is the main glutamate transporter participating to the synaptic transmission[98].
	Glutamine Synthetase	Abcam (ab73593)	1:500	Glutamine Synthetase is a cytosolic enzyme responsible for the breakdown of glutamate in glutamine after uptake through the EAATs[99].
	ALDH1L1	ThermoFisher (14-9595-82)	1:250	Aldehyde dehydrogenase 1 L11 (ALDH1L1) is a pan-astrocyte enzyme involved in folic acid metabolism[100].
**T Cells**	CD4	Dako (IR649)	RTU	CD4 is involved in the recognition of MHC class II molecules and is primarily expressed by the subpopulation of T lymphocytes known as T helper cells[101].
	CD8	Dako (M7103)	RTU	CD8 is a heterodimeric glycoprotein (alpha and beta subunits) that functions in conjunction with the T cell receptor in the recognition of MHC class I/peptide complexes, playing a role in the process of T-cell mediated killing. Thus, the protein mostly identifies the cytotoxic T cells[102].

RTU = ready-to-use concentration as supplied by the manufacturer (Dako)

Supplementary Table 3: (A) Quantification of the pTau markers in the different groups (% protein load) and of the cortical integrity. (B) Representation of the abundance of the pTau staining across disease *vs.* controls, using the median value as reference. Colour code include red: significant increase and high expression; yellow: significant increase and moderate expression; green: significant increase and low expression; blue: no significant difference.

**A**	**Control**	**FTLD-MAPT**	**PiD**	**PSP**	***P* value***
**AT8**	0.010 (0.005-0.017)	0.396 (0.119-1.188)	0.710 (0.236-2.399)	0.058 (0.034-0.259)	*H*(3)=74.38 *P*<0.0001
**AT100**	0.021 (0.001-0.061)	0.391 (0.090-1.158)	0.897 (0.297-1.611)	0.063 (0.025-0.224)	*H*(3)=60.18) *P*<0.0001
**CP13**	0.003 (0.002-0.012)	0.137 (0.031-0.377)	0.473 (0.132-1.300)	0.029 (0.010-0.128)	*H*(3)=63.13 *P*<0.0001
**pThr181**	0.110 (0.024-0.248)	1.298 (0.289-2.737)	2.351 (0.712-3.986)	0.349 (0.072-0.767)	*H*(3)=53.91 *P*<0.0001
**pSer356**	0.013 (0.003-0.073)	4.593 (0.296-12.920)	3.199 (1.015-5.482)	0.150 (0.085-0.606)	*H*(3)=77.63 *P*<0.0001
**pSer396**	0.013 (0.005-0.036)	0.140 (0.008-0.296)	0.456 (0.112-2.696)	0.130 (0.030-0.693)	*H*(3)=44.79 *P*<0.0001
**PHF1**	0.005 (0.002-0.011)	0.012 (0.002-0.124)	0.145 (0.025-0.824)	0.026 (0.010-0.099)	*H*(3)=54.73 *P*<0.0001
**Tau-2**	0.013 (0.006-0.028)	0.050 (0.008-0.081)	0.088 (0.023-0.337)	0.022 (0.006-0.100)	*H*(3)=16.77 *P*<0.0001
**Cortical integrity**	85.87±8.883	78.68 ±8.300	75.37±9.493	84.62±1.163	*F*(3,136)=0.230 *P*<0.0001

Data presented as median (interquartile ranges) or mean ± standard deviation, based on their distribution.
*Kruskal-Wallis test presented as *H*(df), *P* value; ANOVA test presented as *F*(DFn, DFd), *P* value.
FTLD-MAPT = frontotemporal lobar degeneration with tau mutation; PiD: Pick's disease; PSP = Progressive Supranuclear Palsy.

**B**	**AT8**	**AT100**	**CP13**	**pThr181**	**pSer356**	**pSer396**	**PHF1**	**Tau2**
**FTLD-MAPT**								
**PiD**								
**PSP**								

FTLD-MAPT = frontotemporal lobar degeneration with tau mutation; PiD: Pick's disease; PSP = Progressive Supranuclear Palsy

Supplementary Table 4: Quantification of the glial markers in the different groups (% protein load).

	**Control**	**FTLD-MAPT**	**PiD**	**PSP**	***P* value***
**Iba1**	1.179 (0.432-1.845)	1.413 (0.827-3.205)	0.861 (0.292-1.723)	0.981 (0.523-1.693)	*H*(3)=2.867 *P*=0.413
**HLA-DR**	0.206 (0.0628-0.550)	0.130 (0.010-0.317)	0.202 (0.0498-1.240)	0.2873 (0.107-0.741)	*H*(3)=4,323 *P*=0.229
**CD68**	0.103 (0.057-0.132)	0.066 (0.0168-0.150)	0.130 (0.041-0.281)	0.101 (0.058-0.192)	*H*(3)=2.162 *P*=0.540
**CD64**	2.010 (0.846-3.509)	2.548 (0.502-6.222)	1.472 (0.925-3.245)	1.577 (0.590-3.016)	*H*(3)=0.887 *P*=0.829
**CD32a**	1.427 (0.362-2.590)	0.758 (0.116-1.538)	0.966 (0.140-2.557)	1.153 (0.179-2.631)	*H*(3)=2.746 *P*=0.433
**CD16**	0.166 (0.0721-0.740)	0.841 (0.339-2.380)	0.366 (0.158-1.930)	0.263 (0.093-0.859)	*H*(3)=11.26 *P*=0.0104
**GFAP**	6.662 (3.030-13.560)	11.490 (3.539-22.570)	17.310 (6.817-27.150)	7.546 (2.907-13.730)	*H*(3)=13.66 *P*=0.0034
**ALDH1L1**	0.680 (0.257-1.644)	0.809 (0.037-1.863)	0.858 (0.201-1.890)	0.685 (0.311-1.935)	*H*(3)=0.4653 *P*=0.9264
**Glu Syn**	16.300 (8.640-25.090)	3.330 (0.0320-27.110)	7.673 (0.946-18.550)	18.570 (12.650-32.990)	*H*(3)=13.72 *P*=0.0033
**EAAT2**	16.740 (9.758-25.940)	12170 (2.647-16.130)	4.580 (1.807-14.270)	11.500 (6.832-13.760)	*H*(3)=11.63 *P*=0.0033

Data presented as median (interquartile ranges) based on their nn-parametric distribution.
*Kruskal-Wallis test presented as *H*(df), *P* value.
FTLD-MAPT = frontotemporal lobar degeneration with tau mutation; PiD: Pick’s disease; PSP = Progressive Supranuclear Palsy.

Supplementary Table 5. Quantification of the parenchymal and perivascular CD4+ and CD8+ T cells (per $10mm^2$) recruited in the grey matter across the different groups

	**Control**	**FTLD-MAPT**	**PiD**	**PSP**	***P* value***
**Parenchymal CD4+ T cells**	0.626 (0.000-1.144)	1.399 (0.188-2.057)	1.199 (0.455-3.554)	0.667 (0.000-1.755)	*H*(3)=11.16 *P*=0.0109
**Perivascular CD4+ T cells**	0.388 (0.000-1.367)	0.217 (0.000-1.623)	2.971 (0.000-8.200)	1.302 (0.000-2.375)	*H*(3)=9.78 *P*=0.0205
**Parenchymal CD8+ T cells**	3.687 (1.433-11.820)	3.372 (2.144-12.120)	6.604 (1.651-41.800)	3.11 (1.421-6.998)	*H*(3)=5.662 *P*=0.1293
**Perivascular CD8+ T cells**	45.118 (23.588-74.367)	79.158 (47.640-143.190)	69.859 (25.108-213.960)	34.634 (20.893-62.903)	*H*(3)=9.462 *P*=0.0237

Data presented as median (interquartile ranges) based on their non-parametric distribution.
*Kruskal-Wallis test presented as *H*(df), *P* value.
FTLD-MAPT = frontotemporal lobar degeneration with tau mutation; PiD: Pick's disease; PSP = Progressive Supranuclear Palsy

Supplementary Figure 1. Illustrations of pathological tau features previously characterised and seen in our (A-D) PiD and (E) PSP cohorts. PiD = Pick's Disease, PSP = Progressive Supranuclear Palsy. Illustrations of the pTau antibodies in controls. Haematoxylin counterstaining. Scale bar = 50μm

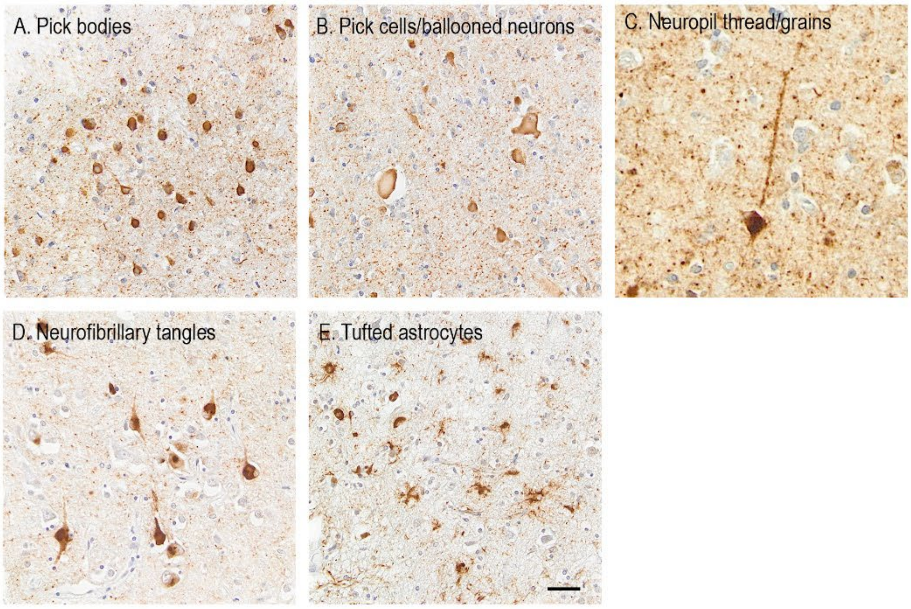

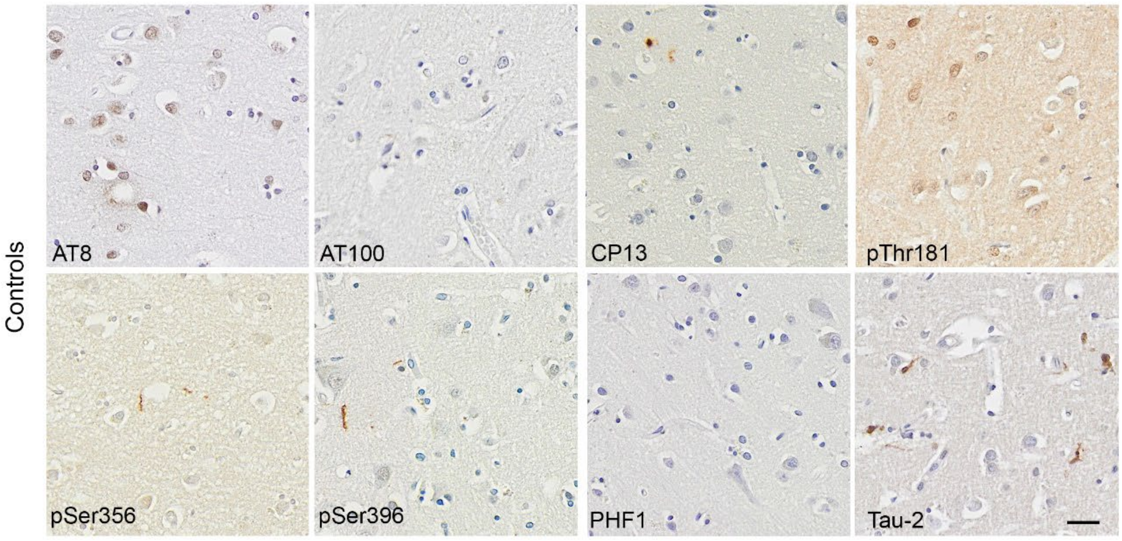

Supplementary Figure 2. Cytokine expression detected using Meso Scale Discovery (MSD) V-Plex Cytokine Panel-1 (human) kit. Graphs show individuals cases and median values. Analysis was carried out by Kruskall-Wallis test. No significant differences were found. PiD = Pick's Disease, PSP = Progressive Supranuclear Palsy

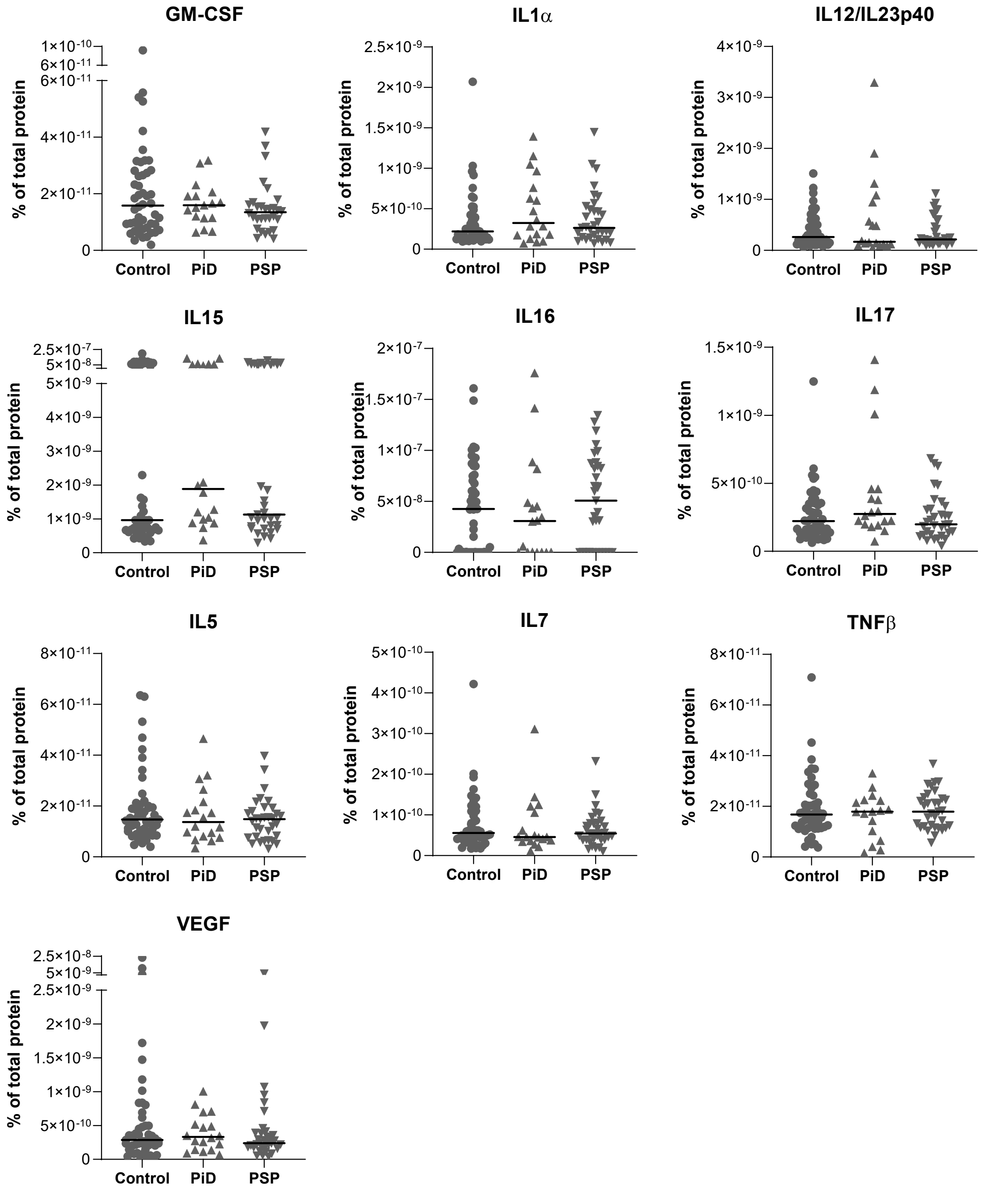

Supplementary Figure 3. **P**ro-inflammatory proteins measured using Meso Scale Discovery (MSD) V-Plex Proinflammatory Panel-1 (human) kit. Graphs show individuals cases and median values. Analysis was carried out by Kruskal-Wallis test. No significant differences were found. PiD = Pick's Disease, PSP = Progressive Supranuclear Palsy.

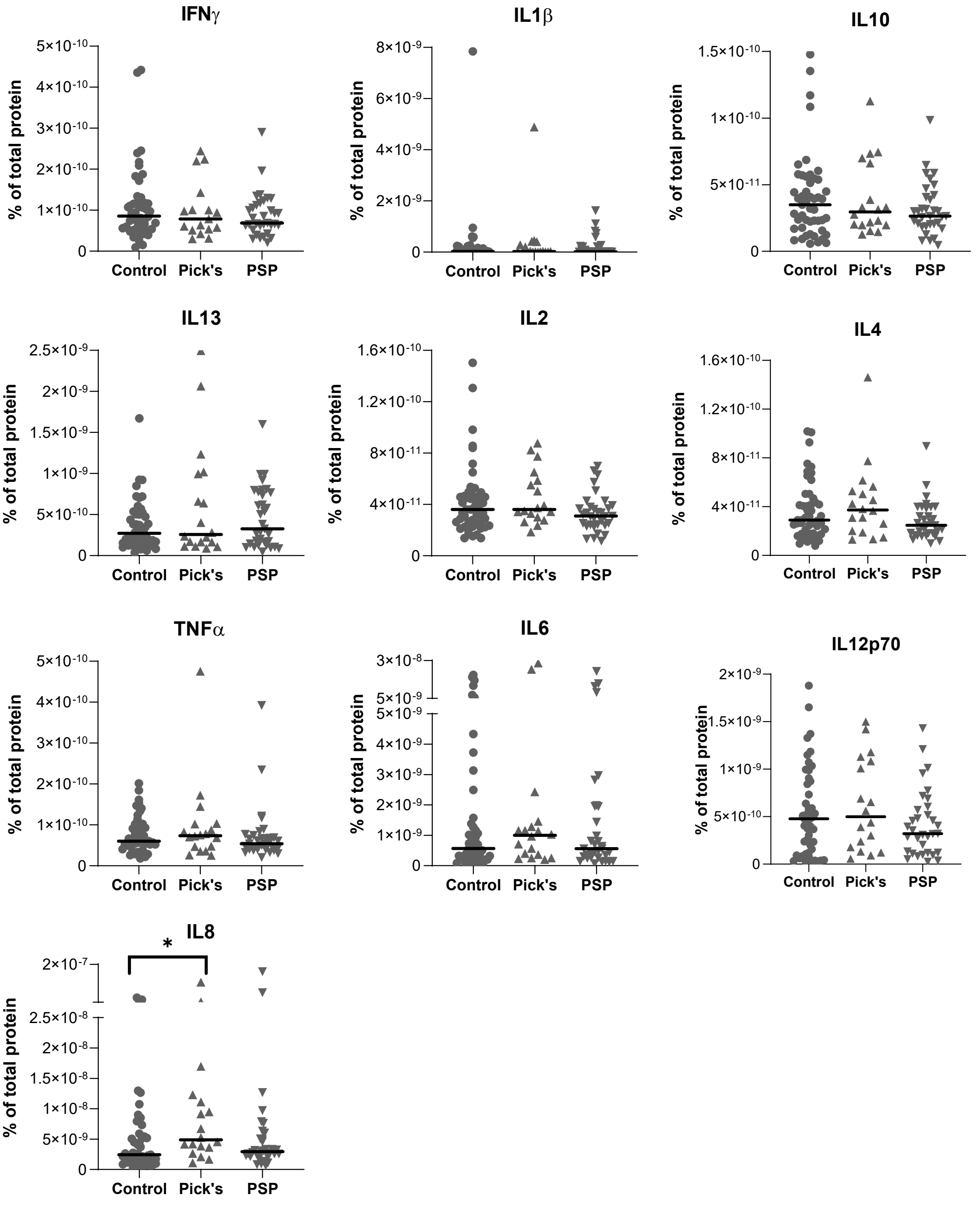

Supplementary Figure 4. Graphs of unaltered chemokine markers expressed in the brain detected using Meso Scale Discovery (MSD) V-Plex Chemokine Panel-1 (human) kit. PiD = Pick's Disease, PSP = Progressive Supranuclear Palsy. Graphs show individuals cases and median values. Analysis was carried out by Kruskall-Wallis test, no significant differences were found – significant difference are shown in Figure 7. PiD = Pick's Disease, PSP = Progressive Supranuclear Palsy

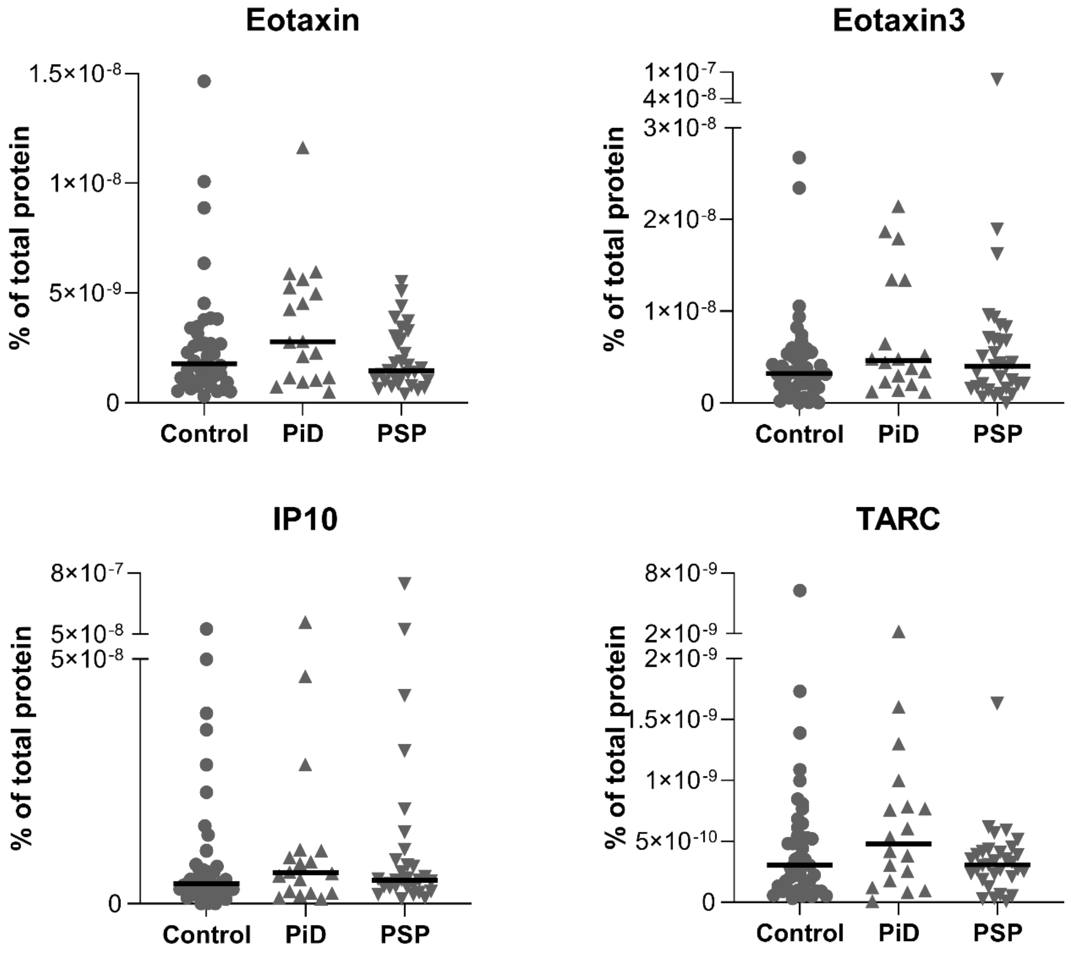

Supplementary Figure 5. Correlations between microglial and tau markers using Spearman's test for non-parametric data presented as: rho value (p value), or Pearson's test presented in italics as: r value (p value). Correlations of tau markers against: A) Iba1, B) HLA-DR, C) CD68, D) CD64/FCγRI, E) CD16/FCγRIII, and F) CD32a/FCγRII. Significant correlation (P<0.001) in bold. PiD = Pick's Disease, PSP = Progressive Supranuclear Palsy

A		Control	FTLD-tau	MAPT	PiD	PSP
Iba1	Tau-2	0.06 (0.6774)	0.08 (0.4679)	-0.11 (0.7329)	0.26 (0.1397)	0.06 (0.7084)
	AT8	0.33 (0.0172)	0.00 (0.9701)	0.20 (0.5431)	0.07 (0.6979)	-0.07 (0.6356)
	AT100	-0.13 (0.3669)	-0.03 (0.7688)	0.17 (0.6039)	0.03 (0.8826)	-0.10 (0.4968)
	CP13	0.28 (0.0473)	-0.08 (0.4401)	0.18 (0.5731)	-0.01 (0.9353)	-0.08 (0.5789)
	Thr181	0.11 (0.4293)	0.04 (0.6979)	0.68 (0.0185)	0.00 (0.9897)	0.03 (0.8501)
	Ser356	0.42 (0.0017)	0.07 (0.4921)	0.55 (0.0667)	0.17 (0.3353)	-0.07 (0.6319)
	Ser396	-0.02 (0.9139)	-0.02 (0.8577)	-0.12 (0.716)	0.14 (0.4217)	0.02 (0.8985)
	PHF1	0.23 (0.1024)	-0.08 (0.4662)	-0.27 (0.4042)	0.12 (0.5006)	0.10 (0.5151)

B		Control	FTLD-tau	MAPT	PiD	PSP
HLA-DR	Tau-2	0.27 (0.0573)	**0.45 (<0.0001)**	**0.73 (0.0096)**	**0.62 (0.0001)**	**0.38 (0.0091)**
	AT8	0.16 (0.2629)	0.27 (0.0111)	0.50 (0.0989)	0.32 (0.073)	**0.44 (0.0028)**
	AT100	-0.12 (0.3799)	0.22 (0.0348)	0.42 (0.1767)	**0.53 (0.0065)**	0.18 (0.2309)
	CP13	0.01 (0.9454)	**0.35 (0.0015)**	0.63 (0.0323)	**0.45 (0.0079)**	**0.48 (0.0087)**
	Thr181	0.22 (0.1223)	0.14 (0.1854)	*-0.01 (0.9724)*	0.20 (0.2689)	0.34 (0.0222)
	Ser356	0.11 (0.4267)	0.00 (0.9861)	-0.35 (0.2662)	-0.04 (0.8189)	0.24 (0.1049)
	Ser396	0.13 (0.3731)	**0.58 (<0.0001)**	**0.93 (0.0038)**	**0.55 (0.0052)**	**0.56 (0.0025)**
	PHF1	0.15 (0.2870)	**0.49 (<0.0001)**	**0.82 (0.0019)**	**0.58 (0.0041)**	**0.45 (0.0022)**

C		Control	FTLD-tau	MAPT	PiD	PSP
CD68	Tau-2	**0.49 (0.0049)**	**0.53 (<0.0001)**	**0.85 (0.0009)**	**0.70 (0.0003)**	**0.40 (0.0068)**
	AT8	**0.39 (0.0044)**	**0.41 (0.0002)**	**0.80 (0.0029)**	0.43 (0.0133)	**0.42 (0.0039)**
	AT100	-0.11 (0.4317)	**0.40 (0.0002)**	**0.76 (0.0055)**	**0.55 (0.0054)**	0.36 (0.0142)
	CP13	**0.37 (0.0076)**	**0.44 (0.0001)**	**0.87 (0.0095)**	0.40 (0.0197)	**0.51 (0.0064)**
	Thr181	0.17 (0.2205)	0.19 (0.0806)	0.25 (0.4303)	0.10 (0.5788)	0.20 (0.1941)
	Ser356	0.15 (0.2969)	0.13 (0.2241)	0.20 (0.5280)	-0.14 (0.4217)	0.31 (0.0361)
	Ser396	0.29 (0.0368)	**0.71 (<0.0001)**	**0.73 (0.0096)**	**0.75 (<0.0001)**	**0.69 (<0.0001)**
	PHF1	**0.44 (0.0010)**	**0.59 (<0.0001)**	0.70 (0.0142)	**0.68 (0.0003)**	**0.51 (0.0064)**

D		Control	FTLD-tau	MAPT	PiD	PSP
CD64	Tau-2	0.32 (0.0202)	**0.45 (0.0001)**	**0.81 (0.0022)**	**0.64 (0.0007)**	0.29 (0.0496)
	AT8	0.08 (0.5721)	**0.39 (0.0004)**	0.57 (0.0591)	0.32 (0.0734)	**0.45 (0.0020)**
	AT100	-0.14 (0.3377)	**0.39 (0.0005)**	0.64 (0.0278)	**0.51 (0.0089)**	0.23 (0.1256)
	CP13	0.26 (0.0696)	**0.35 (0.0015)**	0.50 (0.1041)	0.33 (0.0586)	**0.40 (0.0059)**
	Thr181	-0.04 (0.7584)	0.18 (0.0912)	*0.09 (0.7923)*	0.13 (0.4754)	0.20 (0.1875)
	Ser356	0.09 (0.5364)	0.20 (0.0619)	0.16 (0.6192)	0.04 (0.839)	0.19 (0.2126)
	Ser396	0.21 (0.1410)	**0.43 (<0.0001)**	*0.48 (0.1119)*	**0.48 (0.0045)**	**0.45 (0.0020)**
	PHF1	**0.53 (0.0028)**	**0.37 (0.0009)**	0.58 (0.0521)	**0.55 (0.0055)**	0.37 (0.0128)

E		Control	FTLD-tau	MAPT	PiD	PSP
CD16	Tau-2	0.15 (0.2904)	-0.02 (0.8556)	0.53 (0.0794)	-0.18 (0.3094)	-0.15 (0.3251)
	AT8	0.21 (0.1476)	0.22 (0.0404)	0.24 (0.4573)	-0.07 (0.6856)	0.10 (0.5017)
	AT100	-0.08 (0.5925)	0.11 (0.3107)	0.15 (0.6509)	-0.23 (0.1927)	0.01 (0.9247)
	CP13	0.07 (0.6315)	0.24 (0.0253)	0.20 (0.5431)	0.13 (0.4882)	0.04 (0.8006)
	Thr181	0.03 (0.8232)	0.14 (0.1849)	*-0.43 (0.1605)*	0.16 (0.3796)	0.00 (0.9966)
	Ser356	0.23 (0.1075)	0.22 (0.0347)	-0.38 (0.2276)	0.22 (0.2145)	0.12 (0.4186)
	Ser396	-0.02 (0.8688)	0.03 (0.8104)	*0.45 (0.1383)*	-0.14 (0.4414)	-0.05 (0.7668)
	PHF1	0.23 (0.1066)	0.07 (0.534)	0.59 (0.0457)	-0.05 (0.791)	-0.12 (0.4506)

F		Control	FTLD-tau	MAPT	PiD	PSP
CD32a	Tau-2	0.17 (0.2421)	**0.29 (0.0066)**	0.31 (0.331)	0.41 (0.0197)	0.24 (0.1148)
	AT8	0.13 (0.3791)	0.12 (0.2498)	0.34 (0.2762)	0.25 (0.1594)	0.12 (0.437)
	AT100	-0.15 (0.3058)	0.06 (0.6088)	0.15 (0.6353)	0.24 (0.1822)	0.02 (0.9089)
	CP13	0.13 (0.3484)	0.11 (0.2903)	0.17 (0.5881)	0.18 (0.3153)	0.14 (0.3678)
	Thr181	0.11 (0.4631)	-0.12 (0.2784)	*-0.14 (0.6624)*	-0.12 (0.5148)	0.01 (0.932)
	Ser356	-0.11 (0.4286)	-0.2 (0.0623)	-0.27 (0.4042)	-0.21 (0.246)	-0.23 (0.138)
	Ser396	0.22 (0.1180)	0.23 (0.0304)	*0.46 (0.1336)*	0.36 (0.0412)	0.11 (0.4912)
	PHF1	0.17 (0.2394)	0.19 (0.076)	0.31 (0.3194)	0.41 (0.021)	0.05 (0.7400)

Supplementary Figure 6. Correlations between astrocyte and tau markers using Spearman's test for non-parametric data presented as: rho value (p value), or Pearson's test presented in italics as: *r value (p value)*. Correlations of tau markers against: A) GFAP, B) ALDH1L1, C) Glutamine Synthetase (GS), and D) EAAT2/GLT-1. Significant correlation (*P*<0.001) in bold. PiD = Pick's Disease, PSP = Progressive Supranuclear Palsy

A		Control	FTLD-tau	MAPT	PiD	PSP
GFAP	Tau-2	0.16 (0.2553)	**0.38 (0.0010)**	0.20 (0.5280)	**0.52 (0.0021)**	0.21 (0.1595)
	AT8	0.29 (0.0361)	**0.41 (0.0006)**	0.15 (0.6353)	0.34 (0.0555)	0.35 (0.0193)
	AT100	-0.09 (0.5116)	**0.36 (0.0019)**	0.08 (0.8004)	0.38 (0.0275)	0.26 (0.0856)
	CP13	0.33 (0.0193)	**0.40 (0.0006)**	0.22 (0.499)	0.15 (0.394)	**0.47 (0.0100)**
	Thr181	0.12 (0.3982)	0.23 (0.0282)	*-0.06 (0.8535)*	-0.08 (0.6425)	0.13 (0.4013)
	Ser356	0.25 (0.0730)	0.18 (0.0973)	-0.08 (0.8004)	-0.20 (0.2672)	0.18 (0.2306)
	Ser396	0.16 (0.2771)	**0.58 (<0.0001)**	*0.44 (0.1543)*	**0.52 (0.0020)**	**0.51 (0.0092)**
	PHF1	**0.39 (0.0049)**	**0.52 (<0.0001)**	0.37 (0.2367)	**0.52 (0.0025)**	**0.50 (0.0092)**

B		Control	FTLD-tau	MAPT	PiD	PSP
ALDH1L1	Tau-2	0.05 (0.7150)	0.04 (0.709)	0.10 (0.7493)	0.35 (0.0563)	-0.15 (0.3209)
	AT8	0.17 (0.2210)	-0.03 (0.7546)	0.25 (0.4303)	0.13 (0.4945)	-0.3 (0.0425)
	AT100	0.00 (0.9799)	-0.11 (0.311)	0.04 (0.9039)	0.09 (0.6292)	-0.26 (0.083)
	CP13	0.08 (0.5925)	-0.07 (0.4945)	0.21 (0.5137)	0.13 (0.5005)	-0.27 (0.0722)
	Thr181	0.24 (0.0910)	-0.18 (0.1008)	-0.68 (0.0185)	-0.11 (0.572)	-0.10 (0.4940)
	Ser356	0.34 (0.0161)	-0.18 (0.0922)	-0.61 (0.0399)	0.12 (0.5357)	-0.19 (0.2036)
	Ser396	0.06 (0.6610)	0.09 (0.4075)	0.48 (0.1154)	0.28 (0.1269)	-0.18 (0.2311)
	PHF1	0.00 (0.9842)	0.04 (0.6962)	0.50 (0.1041)	0.28 (0.143)	-0.27 (0.0742)

C		Control	FTLD-tau	MAPT	PiD	PSP
GS	Tau-2	0.23 (0.1004)	0.02 (0.8321)	0.34 (0.2869)	**0.56 (0.0006)**	-0.12 (0.4501)
	AT8	-0.07 (0.6193)	-0.07 (0.5095)	0.01 (0.9739)	0.26 (0.1482)	0.11 (0.4763)
	AT100	0.13 (0.3744)	-0.15 (0.1616)	-0.11 (0.7329)	0.20 (0.2764)	0.15 (0.3154)
	CP13	0.18 (0.2057)	-0.11 (0.3087)	0.10 (0.7664)	0.10 (0.5724)	0.08 (0.5985)
	Thr181	0.18 (0.2135)	-0.26 (0.0156)	-**0.76 (0.0055)**	-0.01 (0.9617)	0.12 (0.4446)
	Ser356	-0.17 (0.2153)	-**0.4 (0.0006)**	-**0.83 (0.0016)**	-0.21 (0.2305)	0.07 (0.6534)
	Ser396	-0.04 (0.7915)	0.26 (0.0121)	0.70 (0.0142)	0.44 (0.0101)	0.28 (0.0708)
	PHF1	0.18 (0.1961)	0.16 (0.1479)	0.55 (0.0667)	0.34 (0.0559)	0.16 (0.3061)

D		Control	FTLD-tau	MAPT	PiD	PSP
EAAT2	Tau-2	0.20 (0.1506)	0.21 (0.0491)	0.70 (0.0142)	0.10 (0.5686)	**0.42 (0.0040)**
	AT8	0.22 (0.1120)	-0.07 (0.4861)	0.37 (0.2367)	0.00 (0.9941)	0.13 (0.3892)
	AT100	-0.13 (0.3494)	-0.13 (0.226)	0.31 (0.331)	-0.07 (0.6993)	0.05 (0.7616)
	CP13	**0.40 (0.0032)**	-0.09 (0.3841)	0.25 (0.4303)	-0.06 (0.7479)	0.16 (0.2943)
	Thr181	0.27 (0.0550)	-0.19 (0.0758)	-0.32 (0.3085)	-0.20 (0.2551)	0.22 (0.1527)
	Ser356	0.24 (0.0906)	-0.06 (0.5589)	-0.21 (0.5137)	0.06 (0.7549)	0.29 (0.0508)
	Ser396	0.12 (0.4120)	0.14 (0.2017)	0.69 (0.0155)	0.17 (0.3516)	0.24 (0.1187)
	PHF1	0.23 (0.1032)	0.06 (0.6106)	0.57 (0.0556)	0.06 (0.7589)	0.14 (0.3557)

Supplementary Figure 7. Correlations between CD8+ T cells and A) tau markers, B) microglial markers and C) astrocyte markers. Correlations for non-parametric data were carried out using Spearman's test presented as: rho value (p value). Parametric data were analysed with Pearson's test presented in italics as: *r value (p value).* Significant correlations (*P*<0.01) are in bold. PiD = Pick's Disease, PSP = Progressive Supranuclear Palsy

A	Control	FTLD-tau	MAPT	PiD	PSP
Tau-2	**0.41 (0.0025)**	**0.43 (<0.0001)**	-0.2 (0.528)	**0.47 (0.0059)**	**0.42 (0.0045)**
AT8	0.09 (0.544)	**0.46 (<0.0001)**	0.13 (0.6832)	0.32 (0.0651)	**0.42 (0.0045)**
AT100	-0.04 (0.797)	**0.42 (<0.0001)**	0.03 (0.9388)	0.32 (0.0693)	0.33 (0.0247)
CP13	0.24 (0.0906)	**0.45 (<0.0001)**	-0.08 (0.8004)	0.27 (0.1223)	**0.51 (0.0007)**
Thr181	0.01 (0.9575)	**0.41 (<0.0001)**	0.13 (0.6999)	0.1 (0.5724)	0.34 (0.0214)
Ser356	-0.01 (0.9502)	**0.39 (0.0002)**	0.3 (0.3424)	0.1 (0.5661)	**0.42 (0.0045)**
Ser396	0.35 (0.0118)	**0.42 (<0.0001)**	-0.39 (0.2097)	0.41 (0.0174)	**0.56 (<0.0001)**
PHF1	0.26 (0.0704)	**0.40 (<0.0001)**	-0.46 (0.134)	**0.47 (0.0069)**	**0.58 (<0.0001)**

B	Control	FTLD-tau	MAPT	PiD	PSP
Iba1	0.16 (0.261)	0.24 (0.0232)	0.31 (0.331)	0.29 (0.1019)	0.16 (0.2859)
CD68	0.25 (0.078)	**0.31 (0.003)**	-0.28 (0.3789)	0.43 (0.0115)	**0.42 (0.0045)**
HLADR	0.29 (0.0395)	0.24 (0.0236)	-0.4 (0.201)	0.37 (0.0351)	**0.43 (0.0045)**
CD64	0.16 (0.2675)	**0.35 (0.0008)**	-0.08 (0.8171)	0.48 (0.0046)	0.31 (0.0405)
CD16	-0.09 (0.5341)	0.05 (0.6646)	-0.01 (0.9739)	-0.03 (0.8899)	0.02 (0.9145)
CD32a	0.28 (0.047)	0.25 (0.0191)	0.42 (0.1767)	0.29 (0.1056)	0.27 (0.0748)

C	Control	FTLD-tau	MAPT	PiD	PSP
GFAP	0.16 (0.2737)	**0.51 (<0.0001)**	-0.43 (0.1689)	**0.61 (0.0017)**	**0.54 (0.0006)**
ALDH1L1	0.08 (0.5811)	0.12 (0.2698)	0.03 (0.9388)	0.24 (0.2005)	0.05 (0.7316)
GluSyn	0.22 (0.1277)	-0.01 (0.9028)	-0.22 (0.499)	-0.05 (0.7903)	0.38 (0.0118)
EAAT2	0.23 (0.111)	-0.07 (0.5149)	-0.13 (0.6832)	-0.09 (0.611)	0.12 (0.4343)

Supplementary Figure 8. Correlations between CD4+ T cells and A) tau markers, B) microglial markers and C) astrocyte markers. Correlations for non-parametric data were carried out using Spearman's test presented as: rho value (p value). Parametric data were analysed with Pearson's test presented in italics as: *r value (p value).* Significant correlations (*P*<0.01) are in bold. PiD = Pick's Disease, PSP = Progressive Supranuclear Palsy

A	Control	FTLD-tau	MAPT	PiD	PSP
Tau-2	0.05 (0.7434)	0.14 (0.1836)	-0.32 (0.3103)	0.00 (0.9867)	0.14 (0.9461)
AT8	0.05 (0.7114)	0.14 (0.1819)	-0.16 (0.6227)	0.08 (0.6399)	0.03 (0.4639)
AT100	0.04 (0.7913)	0.18 (0.0816)	-0.07 (0.8387)	0.10 (0.5661)	0.03 (0.6246)
CP13	0.08 (0.5933)	0.15 (0.1523)	-0.07 (0.8214)	0.09 (0.6253)	0.06 (0.6404)
Thr181	0.01 (0.969)	0.21 (0.0514)	-0.11 (0.7366)	-0.09 (0.6188)	0.2 (0.3005)
Ser356	-0.08 (0.5674)	0.22 (0.0369)	-0.2 (0.5311)	-0.13 (0.4824)	0.31 (0.2457)
Ser396	-0.07 (0.6525)	0.19 (0.0715)	-0.18 (0.5761)	0.06 (0.7423)	0.22 (0.2462)
PHF1	-0.1 (0.4795)	0.16 (0.1422)	-0.15 (0.6459)	0.01 (0.9397)	0.11 (0.2516)

B	Control	FTLD-tau	MAPT	PiD	PSP
Iba1	0.03 (0.8481)	-0.04 (0.6865)	0.04 (0.9164)	-0.01 (0.9647)	-0.03 (0.7525)
CD68	0.04 (0.7603)	0.13 (0.225)	-0.27 (0.3868)	0.15 (0.3909)	0.21 (0.5252)
HLADR	0.01 (0.9306)	0.07 (0.4871)	-0.13 (0.6782)	0.06 (0.7409)	0.17 (0.1842)
CD64	-0.25 (0.0912)	0.01 (0.8907)	-0.38 (0.2239)	0.05 (0.8002)	0.12 (0.0569)
CD16	-0.08 (0.6099)	0.09 (0.376)	-0.54 (0.0757)	0.02 (0.9206)	0.29 (0.1528)
CD32a	0.19 (0.1767)	0 (0.987)	0.02 (0.9432)	-0.04 (0.8219)	0.13 (0.095)

C	Control	FTLD-tau	MAPT	PiD	PSP
GFAP	-0.15 (0.3043)	0.11 (0.312)	-0.3 (0.3383)	0.22 (0.2224)	-0.11 (0.7511)
ALDH1L1	0.09 (0.5369)	0.21 (0.0488)	0.14 (0.6546)	0.06 (0.7623)	0.4 (0.0593)
GluSyn	0.04 (0.7614)	-0.1 (0.3461)	-0.12 (0.7034)	-0.19 (0.2883)	0.22 (0.5383)
EAAT2	-0.05 (0.7587)	-0.18 (0.0843)	-0.25 (0.4254)	-0.24 (0.1736)	0.07 (0.3907)
